# Metal–Polymer Heterojunction in Colloidal-Phase Plasmonic Catalysis

**DOI:** 10.1021/acs.jpclett.1c04242

**Published:** 2022-03-03

**Authors:** Andrea Rogolino, Nathalie Claes, Judit Cizaurre, Aimar Marauri, Alba Jumbo-Nogales, Zuzanna Lawera, Joscha Kruse, María Sanromán-Iglesias, Ibai Zarketa, Unai Calvo, Elisa Jimenez-Izal, Yury P. Rakovich, Sara Bals, Jon M. Matxain, Marek Grzelczak

**Affiliations:** † Galilean School of Higher Education, University of Padova, 35122 Padova, Italy; ‡ EMAT-University of Antwerp, Groenenborgerlaan 171, B-2020 Antwerp, Belgium; ¶ Kimika Fakultatea, Euskal Herriko Unibertsitatea (UPV/EHU) Lardizabal Pasealekua 3, 20018 Donostia-San Sebastián, Spain; § Centro de Física de Materiales (CSIC-UPV/EHU), Paseo Manuel de Lardizabal 5, 20018 Donostia-Sebastián, Spain; ∥ Donostia International Physics Center (DIPC), Paseo Manuel de Lardizabal 4, 20018 Donostia-Sebastián, Spain; ⊥ Ikerbasque, Basque Foundation for Science, Bilbao 48009, Spain

## Abstract

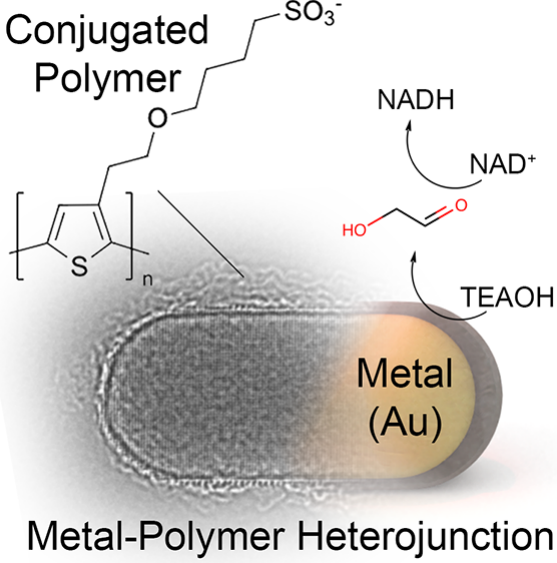

Plasmonic catalysis in the colloidal phase requires robust surface ligands that prevent particles from aggregation in adverse chemical environments and allow carrier flow from reagents to nanoparticles. This work describes the use of a water-soluble conjugated polymer comprising a thiophene moiety as a surface ligand for gold nanoparticles to create a hybrid system that, under the action of visible light, drives the conversion of the biorelevant NAD^+^ to its highly energetic reduced form NADH. A combination of advanced microscopy techniques and numerical simulations revealed that the robust metal–polymer heterojunction, rich in sulfonate functional groups, directs the interaction of electron-donor molecules with the plasmonic photocatalyst. The tight binding of polymer to the gold surface precludes the need for conventional transition-metal surface cocatalysts, which were previously shown to be essential for photocatalytic NAD^+^ reduction but are known to hinder the optical properties of plasmonic nanocrystals. Moreover, computational studies indicated that the coating polymer fosters a closer interaction between the sacrificial electron-donor triethanolamine and the nanoparticles, thus enhancing the reactivity.

Plasmonic catalysis has become a convenient strategy for solar fuel production in which all-metal architectures drive chemical transformation under a wide spectral range of incoming light.^1^ Although the list of photocatalytic processes sustained by plasmonic nanoparticles constantly increases, the mechanisms behind the process remain the subject of intense debate.^2,3^ That is, the contribution of direct photocatalysis involving hot carrier generation by the relaxation of localized surface plasmon resonance (LSPR) has been confronted with thermoplasmonic effect where dissipation of plasmon energy increases the local temperature and thus the rate of a chemical reaction.^4,5^ One of the main reasons for existing discrepancies is not only the diversity of experimental setups and data interpretation but also the complex architecture of plasmonic photocatalysts. In recent years, the emphasis has been put on the design of the plasmonic core, where the size, shape, electronic structure of surface cocatalyst, and the role of surface molecules were assigned as prime attributes. However, the meticulous design of nanoparticles down to the nanometer scale can be sidelined when a photocatalytic reaction involves many particles in both liquid phase or solid substrate, where tiny alteration of interparticle distances—due to aggregation or sintering—shifts the balance from photo- and thermo-catalysis.^4,6^ Regardless of the true mechanism behind plasmonic catalysis, the experimental data indicate that these nanomaterials bring unprecedented opportunities in the development of so-called plasmonic photosynthesis.^7,8^ Certainly, the progress in plasmonic photocatalysis requires not only an assortment of nanoparticles with well-controlled morphology or crystal structure but also universal surface chemistry that enables electron transfer, prevents the particles from aggregation, offers incorporation of functional groups, and ensures their compatibility with eco-friendly solvents.

The quest for a universal molecular shell in plasmonic catalysis in the liquid phase stems from the fact that cationic surfactant molecules, which direct the shape during nanoparticle growth, inhibit the photocatalytic activity of these nanoparticles through a tight interaction with the metal surface.^9−11^ Macromolecular stabilizers such as amphiphilic polymers (PVP,^12,13^ polydopamine^14^) or short alkanethiol molecules^15^ have been proposed as an inert molecular shell in plasmonic catalysis. These molecules, however, suffer from the poor stabilizing ability of typical anisotropic nanoparticles (rods, cubes, and bipyramids) synthesized in the presence of cationic surfactants. Recently, the research community took advantage of conjugated polymers that resulted in convenient stabilizer of metallic nanoparticles,^16−19^ rendering hybrid systems with unprecedented properties, such as optical switches,^20−24^ plasmonic pixels,^25^ printable inks,^26^ therapeutic agents,^27^ and biosensors.^28,29^ These examples show that the rational marriage of metal nanoparticles and conjugated polymers enables hybrid nanostructures of properties inaccessible to the constituting components. We hypothesized that the combination of conjugated polymers and plasmonic nanoparticles enable the metal–polymer heterojunction, which apart from preventing particles aggregation, can provide selective interaction of plasmonic core with the reagents. In fact, to date, the use of conjugated polymers as the molecular interface in the plasmonic catalysis has yet to be achieved.

Herein, we show that water-soluble conjugated polymers can promote the formation of a metal–organic heterojunction on gold nanoparticles of different shapes, including spheres, cubes, rods, and bipyramids. The in situ spectroscopy, ex situ advanced electron microscopy, and numerical calculations revealed that the polymer molecules, by forming covalent bonds with the metal surface, can form a tight nanostructured shell. In addition, the physical properties of a metal–polymer hybrid differ from those of their constituting building blocks as the plasmonic core is subjected to electron doping and the excited state of polymer shell exhibits a shorter lifetime. We used the colloidally stable polymer-coated gold nanoparticles in the photocatalytic regeneration of cofactor molecules, showing that the conjugated polymers are a convenient alternative for transition-metal surface cocatalysts (Figure 1).

**Figure 1:**
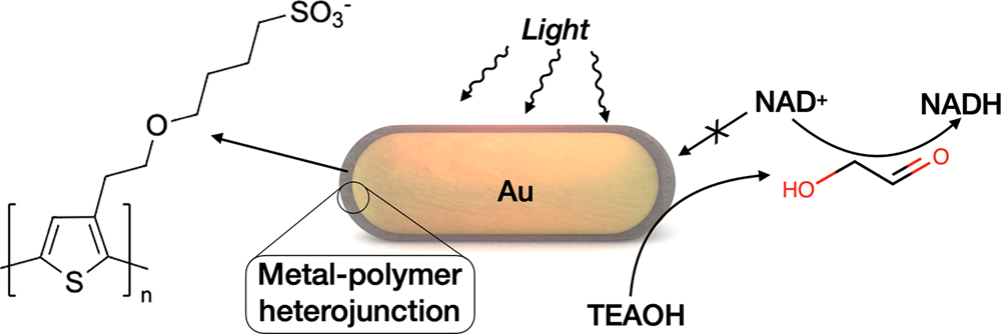
Water-soluble conjugated polymer covering gold nanoparticles promotes selective oxidation of electron donor molecules in the presence of light.

*Ligand Exchange*. We selected poly[2-(3-thienyl)-ethyloxy-4-butylsulfonate] (PTEBS) as a model polymer for metal–organic heterojunction. PTEBS exhibits good solubility in water at relatively high concentrations (0.6 mg/mL) and is capable of replacing the cationic surfactant (i.e., cethyltrimethylammonium bromide (CTAB)) from the surface of gold nanorods.^26^ To show the universal character of PTEBS as a capping agent, we expanded the list of possible nanoparticle shapes, including nanocubes, bipyramids, and rods (Figure S1), which were all initially stabilized with CTAB. Upon ligand exchange, the localized surface plasmon band blueshifted by 4, 7, and 13 nm for the cubes, bipyramids, and nanorods, respectively (Figure 2a). Typically, the replacement of the CTAB bilayer with a polymer leads to a redshift of the localized surface plasmon resonance (LSPR) as a result of the increased refractive index close to the particle surface,^30^ a scenario one would expect here. Eventual chemical etching of metallic gold during ligand exchange would suggest blueshift of the LSPR, but such an option was ruled out because TEM analysis confirmed the invariance of length and width (Figure 2b).

**Figure 2:**
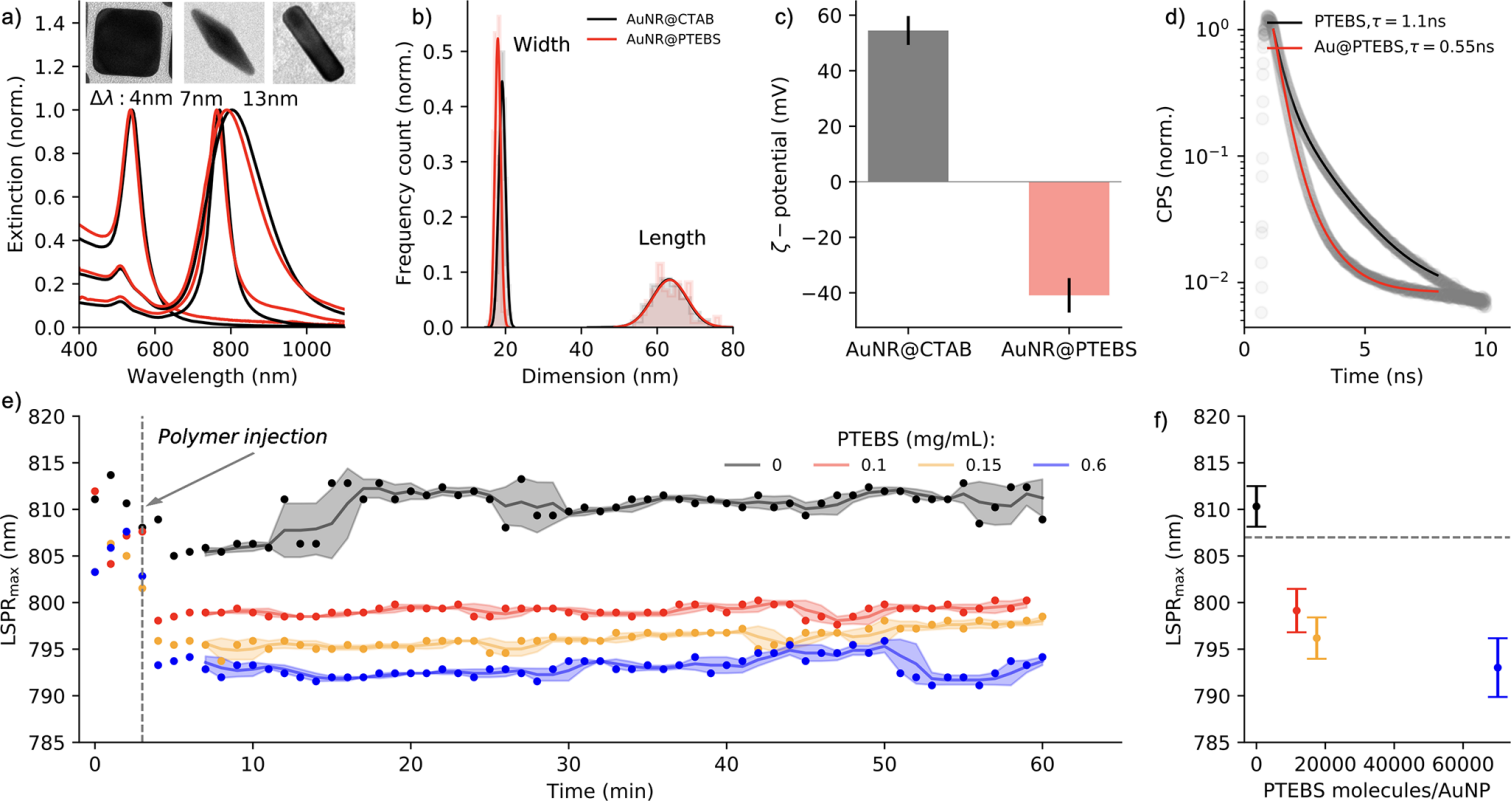
Ligand exchange. (a) UV–vis–NIR spectra of initial and polymer-stabilized gold nanoparticles. (b) Size distribution of widths and lengths of gold nanorods before and after ligand exchange. (c) Reversal of ζ-potential during ligand exchange. (d) Fluorescence decay of free PTEBS and AuPTEBS. (e) Time-dependent change of the maximum of LSPR during ligand exchange for different PTEBS concentrations, showing blueshift of LSPR with increasing PTEBS concentration. (e) Position of the maximum of LSPR versus the number of PTEBS molecules per gold nanorod.

We hypothesized that the electron-donating character of the PTEBS is responsible for the consistent blueshift of the plasmon band upon ligand exchange. We performed time-dependent UV–vis–NIR characterization of the sample during ligand exchange for different concentrations of PTEBS (Figure 2e). Fast addition of PTEBS solution (from 0 to 0.6 mg/mL) to the colloidal dispersion of gold nanorods caused an abrupt blueshift of the LSPR that lasted for an extended period of time (Figure 2f). The blueshift of LSPR as a function of added polymer shows a nonlinear relationship with the number of polymer molecules per gold nanorod, reaching a plateau at higher polymer concentrations. Mulvaney and co-workers have estimated that a blueshift of LSPR by 11 nm requires an injection of ∼80 000 electrons to an individual gold nanorod in an electrochemical process.^31,32^ In our case, we observed a similar shift of LSPR for the highest amount of polymer in solution, that is, ∼70 000 PTEBS molecules per particle. Each PTEBS molecule (70 000 g/mol) contains 245 monomers capable of donating at least one electron through a lone electron pair in sulfur.^33^ Therefore, during ligand exchange, nanoparticles are exposed to an electron-rich environment where the electron transfer to metallic gold is favored by the formation of Au–S covalent bonds and the interaction of conjugated thiophene electrons with Au d-orbitals.^34^ The electron transfer can continue even after the formation of a compact polymeric shell, especially if there is an excess of free polymer in solution (∼16 molecules per nm^2^ of metallic gold) and assuming a conductive character of the polymer shell.^26^ The reversal of ζ-potential values before and after the ligand exchange additionally confirmed the presence of polymer on the surface of gold nanoparticles (Figure 2c). Upon the functionalization of gold nanoparticles with PTEBS, the lifetime of excited states of the polymer decreased from 1.1 to 0.55 ns, suggesting fluorescence quenching via energy transfer^35^ (Figure 3d). Overall, the formation of a polymer–metal heterojunction mutually alters the properties of the plasmonic core and polymer shell.

**Figure 3:**
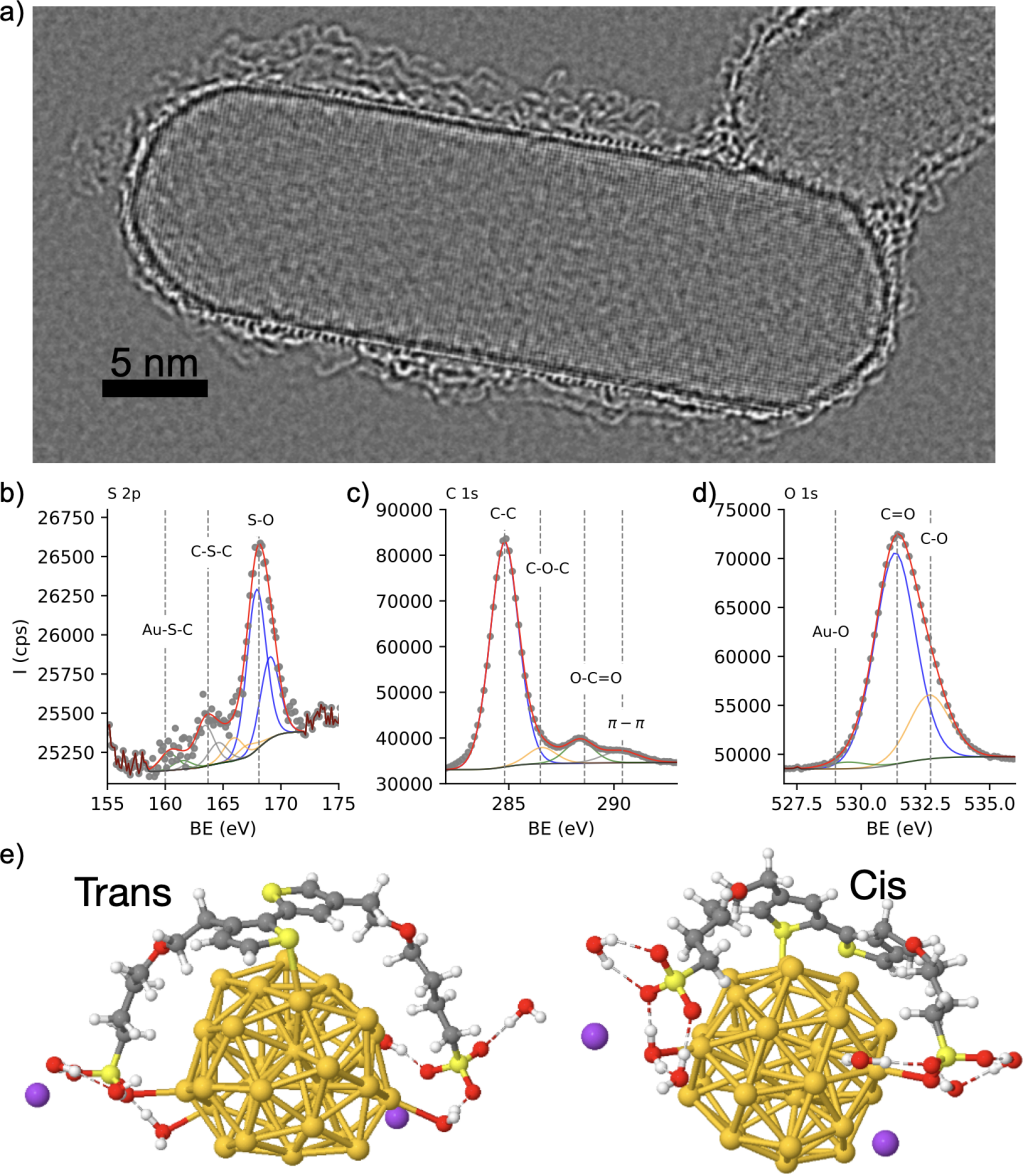
Metal–polymer heterojunction. (a) Phase image by exit wave reconstruction of individual nanorods coated with PTEBS showing both the monocrystalline structure of the nanoparticle and the polymer shell. (b–d) XPS analysis of AuPTEBS showing the interaction of polymer with gold through covalent bond Au–S–C in panel b and the formation of π–π stacks in polymer chains (c). (e) Calculated structure of gold cluster (34 atoms) and TEBS dimers in cis and trans conformation showing that sulfur (thiophene) and oxygen (sulfonate) are the primary anchoring points to metallic gold.

*Structure of Polymer–Metal Heterojunction*. To visualize the crystal structure of the nanocrystal and the polymer on its surface, a focal series of high-resolution TEM images were acquired followed by an exit wave reconstruction in order to retrieve the phase image.^36^ The obtained phase images showed that polymer chains form a ∼2 nm thick shell (Figure 3a), corroborating previous results by Kraus and co-workers.^26^ Interestingly, the anchoring of PTEBS to anisotropic nanocrystals leads to a fairly homogeneous coating, regardless of the local curvature of the surface. Such a scenario is not within the reach of thiol-terminated polymers of a similar molecular weight, which form radially distributed brushes preferentially at the tips, favoring formation of patches and often compromising the colloidal stability of the nanoparticles.^37^ X-ray photoemission spectroscopy (XPS) further confirmed that the metal–polymer interface is maintained through the Au–S–C (S 2p, 160 eV) from thiophene moiety and Au–O bonds (O 1s, 529 eV) from sulfonate terminal groups (Figure 3b,d). The formation of π–π interactions between the thiophene rings (C 1s, 290.5 eV in Figure 3c) suggests that the polymer can form organized structures in either face-on or edge-on fashion.^26,38^ It is reasonable to assume, however, that in the liquid phase, the hydrated polymer extends toward the bulk solution, resulting in a less compact structure. The hydrodynamic diameter of spherical nanoparticles increased by ∼10 nm after displacing native ligands with PTEBS as confirmed by dynamic light scattering analysis (Figure S2).

To obtain a qualitative view of the polymer–gold interaction, we performed DFT calculations on model systems for both gold nanoparticles and PTEBS (see the Supporting Information, section 5.1). Concretely, gold nanoparticles were modeled using a Au_34_ cluster and PTEBS structures using smaller constituents, such as methyl-tiophene dimers both in cis and trans conformations, methyl sulfonate groups, and complete TEBS dimers in either cis or trans conformation (a detailed description of the models is available in the Supporting Information, section 5.2). The calculations confirmed that the polymer interacts with gold via sulfur atoms of thiophene rings and oxygen atoms of the sulfonate group (Figure 3e, Table 1), suggesting that cooperative interactions ensure a homogeneous coverage of the gold nanoparticle.

**Table 1:** Calculated Interaction Enthalpies for Gold (Au_34_ Cluster) and Polymer Parts, Such as Tiophene Dimers in Cis and Trans Conformations, Sulfonate Chain Models and Full Dimer of PTEBS in Cis and Trans Conformations^a^

Au	*ΔH*	*r*(Au–S_t_)	*r*(Au–S_*s*_)	α (C–S–Au)	ϕ (C–S–S–C)
Au-tiophene (cis)	–33.04	2.66; 3.57		95.6; 97.4	–2.56
Au-tiophene (trans)	–34.25	2.72; 3.28		90.4; 98.4	174.4
Au-sulfonate	–19.68		3.34		
Au-dimer (cis)	–63.84	2.61; 4.39	3.63 (4.57)	94.1; 52.0	17.84
Au-dimer (trans)	–83.74	2.79; 3.41	4.32 (3.63)	95.5; 89.9	–173.3

a In addition, distances between gold and sulfur atoms (both from tiophene, *S*_t_, and sulfonate group, *S*_s_) are given in Å, along with C–S–Au bond angle, and the dihedral angle between tiophene rings, in degrees.

The interaction enthalpies between a gold nanocluster and tiophene dimers fall within the range of 33–34 kcal/mol, while the interaction with the sulfonate group is 20 kcal/mol (Table 1), suggesting that gold interacts more strongly with the tiophene sulfur atom. Nevertheless, the Au–S interatomic distances impose that only one of the sulfur atoms interacts with the nanocluster, thus conserving the ring coplanarity and the π delocalization (the values of the dihedral angle are close to 0 or 180°).

Regarding the gold–TEBS dimer interaction, one would expect the interaction energy values of the PTEBS dimers to approach −73 kcal/mol, the sum of the interaction energies of the constituents. The obtained values, however, are −83 and −63 kcal/mol for the trans and cis conformers, respectively, because of destabilization of the cis dimer during interaction with gold. Indeed, the C–S–S–C dihedral angle (related to the planarity between tiophene rings) in the cis conformation is enlarged from 2.5 to 17.8°, indicating the breaking of the π delocalization over the rings. This is not the case in the trans conformation, which conserves the ring planarity even in the PTEBS dimer model. These results indicate that not all sulfur atoms are capable of binding at the same time to the gold surface, corroborating the information obtained from HRTEM imaging. Additionally, the most stable polymeric structure was determined using PW-DFT along with periodic boundary conditions (section 5.2 in the Supporting Information). In line with the cluster-fragment model, the polymer trans conformation is thermodynamically more stable than the cis conformation. Both periodic and cluster models show the presence of π–π interactions between thiophene groups in the polymer, which is calculated to be around 5 kcal/mol.

Overall, the trans conformation is favored in the isolated polymer and at the interface with gold, favoring both the tiophene–gold interaction and the sulfonate–gold interaction. The DFT calculations point to the possible π–π interaction between thiophene groups in polymer, as is observed in the most stable polymer structure calculated with periodic boundary conditions (see the Supporting Information, section 5.1). We expect that this interface interaction would favor the formation of sulfonate–sulfonate and π–π interactions between polymeric chains similar to the isolated polymer, which would lead to the strong stabilization of the metal–polymer heterojunction, as observed experimentally.

*Photocatalytic Properties*. The photocatalytic activity of AuPTEBS was tested in the light-assisted photoreduction of NAD^+^ to NADH using triethanolamine (TEAOH) as an electron donor. The nonenzymatic photoreduction of NADH is a long-lasting target in photobiocatalysis because it would open up the possibilities for efficient recycling of cofactor molecules in enzymatic cascade reactions of technological relevance and thus lower the cost of a given process.^39−41^ We have previously shown that in semiconductor-based photocatalysis, in the presence of oxygen, TEAOH molecules undergo photodegradation via the aminyl radical producing glycolaldehyde which in turn reduces NAD^+^ to NADH.^42^ We postulate that PTEBS as a molecular interface can enhance the process by favoring the selective interaction of TEAOH with the photocatalyst.

We performed photocatalytic reduction of NAD^+^ to NADH at the steady-state temperature profiles ranging from 20 to 55 °C for dark and light reaction (power density = 200 mW/cm^2^; spectral range = 450–1200 nm) (Figure 4a). The reactor was thermostated, and the temperature of the mixture was monitored in real-time during the light and dark processes (Figure 4b). In the dark, although we observed the evolution of the characteristic band at 340 nm indicating NADH regeneration after 2 h, the rate was temperature-independent (Figures 4c and S3). The gray lines in Figure 4c adopted the same slope, suggesting that in the presence of AuPTEBS the thermal regeneration of NADH remains inhibited. Under light conditions, the scenario changed drastically: the regeneration of NADH increased by 8 times as compared to dark conditions. The higher the temperature, the higher the rate of NADH regeneration, giving an activation energy *E*_a_ = 61 kJ/mol (Figure 4d). Interestingly, we observed that above 50 °C, the initially high regeneration rate of NADH was lowered at the later stage of the reaction (60 min). We hypothesized that such a drop in the regeneration is due to the partial oxidation or degradation of reduced NADH. To evaluate the hypothesis we monitored both the change of absorbance at 340 nm and the position of the maximum of LSPR in the mixture containing NADH and AuPTEBS and at temperatures ranging from 30 to 60 °C (Figure S4). We found that with increasing temperature, the absorbance at 340 nm decreases and LSPR blueshifts, indicating the oxidation of the NADH to NAD^+^ at elevated temperatures. The evolution of reduced NADH as a function of incident light power density adopted a linear relationship (Figures 4e and S5), indicating photocatalytic transformation of compounds, because an exponential increase would suggest an Arrhenius-type relationship and thus the involvement of a thermoplasmonic effect.^4^

**Figure 4:**
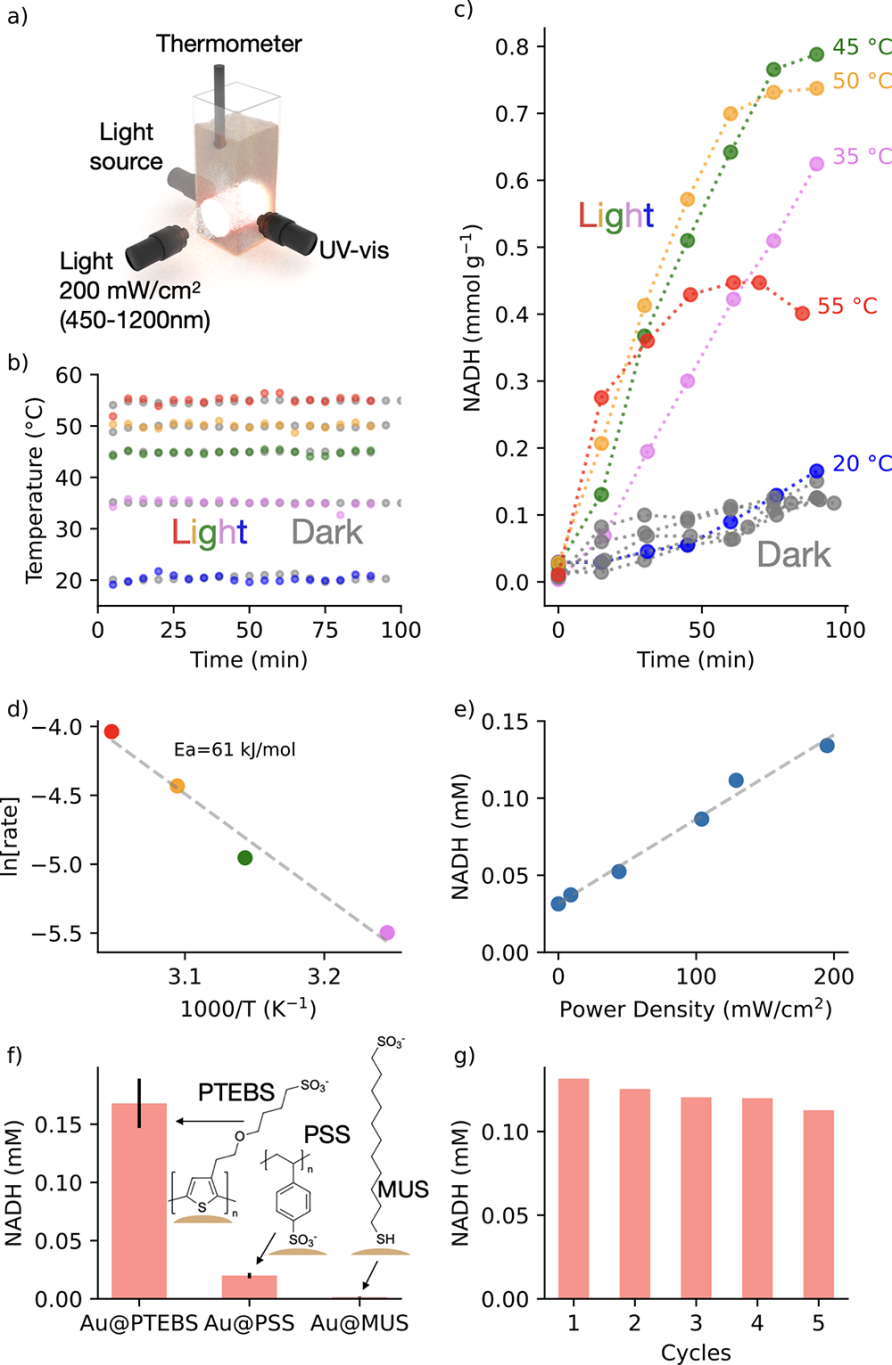
Photocatalytic regeneration of cofactor molecules on AuPTEBS. (a) Scheme of the reactor comprising real-time analytics: temperature and spectroscopy. (b) Steady-state temperature profile under light (colored) and dark (gray) conditions. (c) Time-dependent evolution of NADH at temperatures ranging from 20 to 55 °C under light (colored) and dark (gray) conditions. (d) Arrhenius analysis of the process. (e) Regeneration of NADH as a function of power density. (f) Effect of surface ligand on NADH regeneration. (g) Cyclic regeneration of NADH.

To show the advantage of using a conjugated molecular shell we compared the photocatalytic performance of gold nanorods coated with other nonconjugated molecular systems. We selected a polymer comprising sulfonate groups, namely, polystyrenesulfonate (PSS) and alkanethiol 11-mercapto-1-undecanesulfonate (MUS) that comprise both thiol and sulfonate functional groups. We observed that the system comprising conjugated polymer outperforms PSS and MUS (Figures 4f and S6), confirming that the conjugated molecular shell in metal–polymer heterojunction allows for efficient electron transfer in redox reactions. Also, a large number of anchoring points in PTEBS polymer ensures the structural integrity of hybrid systems, as confirmed by NADH photoregeneration in five consecutive cycles (Figures 4g and S7). A drop in performance with each cycle was due to the losses of nanoparticles in each centrifugation step. Finally, in a control experiment we showed that PTEBS polymer is unable to catalyze regeneration of cofactor molecules (Figure S8). Overall, these results show that metal–polymer heterojunction makes possible the exclusion of any transition metal (platinum or palladium) playing a role of surface cocatalyst.^11,43,44^

Although the results above indicate a nonthermal mechanism, we cannot neglect the temperature increase close to the particles’ surface under light conditions that is due to the photothermal heating by a large number of particles. Baffou has demonstrated that under continuous irradiation, the temperature from a nanoparticle heat source decays as 1/*r*, with *r* being the radial coordinate.^6^ Thus, for small interparticle distances (from hundreds of nanometers to several micrometers), the collective heating tends to homogenize the temperature on the macroscopic level. The overall increase of temperature (*δT*) is then the sum of local heating of single particles (*δT*^self^) and collective heating from neighbor particles (*δT*^ext^). Equation 1 has been proposed to distinguish the contribution of both regimes,^6^ which takes into account geometrical parameters of a multiparticle system returning a unit-less coefficient:
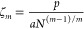
1where *p* is the average interparticle distance, *a* the equivalent diameter of a nanoparticle, *N* the total number of nanoparticles in solution, and *m* the dimensionality coefficient (for a colloidal solution *m* = 3). The collective heat generation becomes dominant when ζ_*m*_ ≪ 1. In our system, *a* = 32 nm^2^, *N* = 3 × 10^11^, and *p* = 2 μm, giving ζ_3_ ≈ 2 × 10^–6^. Thus, because ζ_*m*_ ≪ 1, the collective heating under steady-state radiation can accelerate the oxidation of TEAOH to glycolaldehyde close to the particles surface followed by NADH regeneration at larger distances. However, to monitor the local temperature change, it is necessary to use sophisticated methods such as, for instance, fluorescent thermometry combined with DNA technology.^38^ Note that in our hybrid system, emission from the polymer shell makes possible measurements of the lifetime of excited states (Figure 2d) which in principle is sensitive to the local temperature. Therefore, we postulate that the present system makes it possible to monitor the change of temperature in situ during a photocatalytic process.

Our reaction model states that TEAOH molecules undergo catalytic degradation to glycolaldehyde that in turn reduces the cofactor molecules out of nanoparticles surface.^42^ Such a model requires a preferential interaction of an amine (sacrificial electron donor) with gold than interaction of a cofactor with polymer-coated nanoparticles. To evaluate such a scenario we measured emission spectra of pure polymer mixed with either TEAOH or NAD^+^. With increasing the concentration of TEAOH (from 0 to 0.25 M) the emission of PTEBS (0.1 mg/mL) increased, accompanied by a 12 nm redshift (Figure 5a), suggesting that TEAOH molecules alter the relaxation of excited state of the polymer that is due to the formation of a PTEBS-TEAOH complex. By contrast, the emission of PTEBS remains unchanged when the polymer mixture is exposed to the increasing concentration of NAD^+^ (0–1 mM) (Figure 5b), indicating no interaction between cofactor and polymer, and being quite the opposite for methyl viologen that quenches the emission of oligothiophenes.^45^ The DFT calculations additionally revealed that TEAOH molecules interact with PTEBS preferentially through the sulfonate group (−27.34 kcal/mol, hydrogen bonding) rather than through the thiophene moiety (Figure 5c). Therefore, in liquid phase the polymer anchored to the gold surface can undergo nanostructuring to form hydrophilic (sulfonate-rich) and hydrophobic (thiophene-rich) regions (Figure 5d), where sulfonate groups can play a role of buffer in bringing the ternary amine close to the metallic surface to form a Au–N covalent bond (−36.53 kcal/mol). [Note that TEAOH is able to stabilize gold nanorods (Figure S9), but the nanoparticles aggregate when subjected to photocatalytic mixture.] Therefore, the amphiphilic nature of PTEBS has a beneficial role in terms of colloidal stabilization and catalysis: (i) hydrophobic thiophene moieties strongly bind to the surface of nanoparticles replacing CTAB and preventing aggregation and (ii) hydrophilic sulfonate groups bear negative charges to the surface, as indicated by the reversed value of ζ-potential after ligand exchange, which is likely to improve the docking of triethanolamine on Au and subsequent oxidation to glycolaldehyde. Under light conditions, the decay of surface plasmon resonance to polymer can favor sequential extraction of two electrons from amine to finally liberate glycolaldehyde, which in turn leads to the regeneration of cofactor molecules.

**Figure 5:**
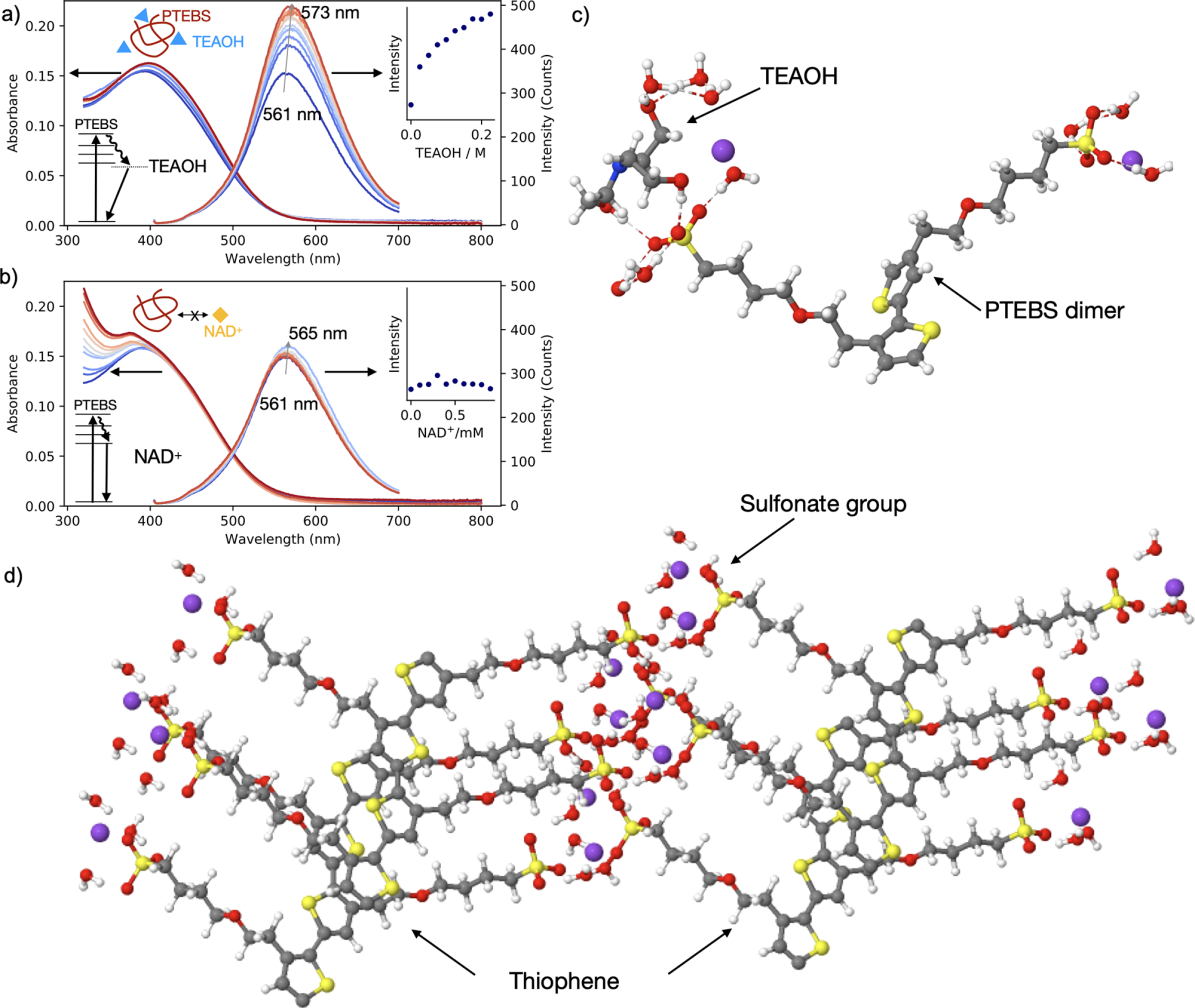
Polymer–reagent interaction. (a and b) Absorbance and emission spectra of (a) PTEBS-TEAOH and (b) PTEBS-NAD^+^ with increasing concentration of both reagents. The PTEBS emission spectra redshift and gain intensity in the presence of TEAOH. The spectra remain unchanged in the presence of NAD^+^ (insets). (c) Calculated stable structure of TEBS dimer and TEAOH showing preferential interaction of TEAOH with sulfonate groups. (d) Calculated structure of PTEBS with visible structuring of thiophene and sulfonate regions.

In summary, the rational combination of polymer and plasmonic particles leads to a hybrid structure in which both components are mutually affected: gold nanocrystals experience strong electron doping, while the polymer particles exhibit faster radiative recombination. For the sake of generality, we showed that polymer can stabilize gold nanoparticles of different shapes that have a cationic surfactant as the native ligand. Detailed structural analysis revealed that the polymer molecules form a homogeneous shell on the surface of gold nanorods and that the structuring of the polymer on the nanoparticles surface is an important ingredient in the design of photocatalytic systems, favoring efficient oxidation of sacrificial molecules. As a proof of concept, we showed that conjugated polymers could successfully replace noble metal cocatalysts in the regeneration of the ubiquitous cofactor NADH. In particular, hydrophilic moieties of polythiophene favor the interaction between the plasmonic nanoparticles and the widely used electron donor triethanolamine. The observed enhanced electron transfer in the metal–polymer heterojunction opens new possibilities in plasmon-assisted reductive catalysis. Bearing in mind the vast diversity of available conjugated polymers, diversity of plasmonic nanoparticles, and facile replacement of native ligands with PTEBS, we foresee that the rational design of metal–polymer heterojunctions presents a new toolbox in plasmonic catalysis without the use of a transition-metal surface cocatalyst. For example, the combination of water-soluble poly(3,4-ethylenedioxythiophene) (PEDOT)^46^ with plasmonic nanoparticles is an attractive strategy for constructing p–n junction architectures down to the level of a few nanoparticles. Therefore, metal–polymer heterojunctions containing a plasmonic core and a water-soluble conjugated polymer shell are strong candidates for a photocatalytic system in the liquid phase.

## Supporting Information Available

Materials and methods; additional characterizations: TEM, DLS, UV–vis; control experiments; detailed description of computational methods (PDF)
Transparent Peer Review report available (PDF)

## References

[ref1] Zhang Y.; Guo W.; Zhang Y.; Wei W. D. Plasmonic Photoelectrochemistry: In View of Hot Carriers. Adv. Mater. 2021, 33, 2006654 10.1002/adma.202006654. 33977588

[ref2] Zhou L.; Swearer D. F.; Zhang C.; Robatjazi H.; Zhao H.; Henderson L.; Dong L.; Christopher P.; Carter E. A.; Nordlander P.; et al. Quantifying Hot Carrier and Thermal Contributions in Plasmonic Photocatalysis. Science 2018, 362, 69–72. 10.1126/science.aat6967. 30287657

[ref3] Dubi Y.; Sivan Y. Hot” Electrons in Metallic Nanostructures—non-thermal Carriers or Heating?. Light Sci. Appl. 2019, 8, 89 10.1038/s41377-019-0199-x. 31645933 PMC6804576

[ref4] Baffou G.; Bordacchini I.; Baldi A.; Quidant R. Simple Experimental Procedures to Distinguish Photothermal From Hot-carrier Processes in Plasmonics. Light Sci. Appl. 2020, 9, 108 10.1038/s41377-020-00345-0. 32612818 PMC7321931

[ref5] Dubi Y.; Un I. W.; Sivan Y. Thermal Effects - An Elternative Mechanism for Plasmon-assisted Photocatalysis. Chem. Sci. 2020, 11, 5017–5027. 10.1039/C9SC06480J. 34122958 PMC8159236

[ref6] Baffou G. Thermoplasmonics: Heating Metal Nanoparticles Using Light; Cambridge University Press: Cambridge, 2017.

[ref7] Jain P. K. Taking the Heat Off of Plasmonic Chemistry. J. Phys. Chem. C 2019, 123, 24347–24351. 10.1021/acs.jpcc.9b08143.

[ref8] Yu S.; Jain P. K. The Chemical Potential of Plasmonic Excitations. Angew. Chem., Int. Ed. 2020, 59, 2085–2088. 10.1002/anie.201914118. 31765516

[ref9] Liu L.; Ouyang S.; Ye J. Gold-Nanorod-Photosensitized Titanium Dioxide with Wide-range Visible-light Harvesting Based on Localized Surface Plasmon Resonance. Angew. Chem., Int. Ed. 2013, 52, 6689–6693. 10.1002/anie.201300239. 23666880

[ref10] Zheng Z.; Tachikawa T.; Majima T. Plasmon-enhanced Formic Acid Dehydrogenation Using Anisotropic Pd–Au Nanorods Studied at the Single-Particle Level. J. Am. Chem. Soc. 2015, 137, 948–957. 10.1021/ja511719g. 25543832

[ref11] Tarnowicz-Staniak N.; Vázquez-Díaz S.; Pavlov V.; Matczyszyn K.; Grzelczak M. Cellulose as an Inert Scaffold in Plasmon-assisted Photoregeneration of Cofactor Molecules. ACS Appl. Mater. Interfaces 2020, 12, 19377–19383. 10.1021/acsami.9b21556. 32253909 PMC7497628

[ref12] Zhai Y.; DuChene J. S.; Wang Y.-C.; Qiu J.; Johnston-Peck A. C.; You B.; Guo W.; DiCiaccio B.; Qian K.; Zhao E. W.; et al. Polyvinylpyrrolidone-induced Anisotropic Growth of Gold Nanoprisms in Plasmon-driven Synthesis. Nat. Mater. 2016, 15, 889–895. 10.1038/nmat4683. 27376686

[ref13] Kim Y.; Smith J. G.; Jain P. K. Harvesting Multiple Electron–hole Pairs Generated Through Plasmonic Excitation of Au Nanoparticles. Nat. Chem. 2018, 10, 763–769. 10.1038/s41557-018-0054-3. 29736005

[ref14] Feng J.-J.; Zhang P.-P.; Wang A.-J.; Liao Q.-C.; Xi J.-L.; Chen J.-R. One-step Synthesis of Monodisperse Polydopamine-coated Silver Core–shell Nanostructures for Enhanced Photocatalysis. New J. Chem. 2012, 36, 148–154. 10.1039/C1NJ20850K.

[ref15] Roy S.; Jain V.; Kashyap R. K.; Rao A.; Pillai P. P. Electrostatically Driven Multielectron Transfer for the Photocatalytic Regeneration of Nicotinamide Cofactor. ACS Catal. 2020, 10, 5522–5528. 10.1021/acscatal.0c01478.

[ref16] Vijayakumar C.; Balan B.; Saeki A.; Tsuda T.; Kuwabata S.; Seki S. Gold Nanoparticle Assisted Self-Assembly and Enhancement of Charge Carrier Mobilities of a Conjugated Polymer. J. Phys. Chem. C 2012, 116, 17343–17350. 10.1021/jp3039253.

[ref17] Han J.; Wang M.; Hu Y.; Zhou C.; Guo R. Conducting Polymer-noble Metal Nanoparticle Hybrids: Synthesis, Mechanism and Application. Prog. Polym. Sci. 2017, 70, 52–91. 10.1016/j.progpolymsci.2017.04.002.

[ref18] Kanelidis I.; Kraus T. The Role of Ligands in Coinage-metal Nanoparticles for Electronics. Beilstein J. Nanotechnol. 2017, 8, 2625–2639. 10.3762/bjnano.8.263. 29259877 PMC5727811

[ref19] Jung I.; Kim M.; Kwak M.; Kim G.; Jang M.; Kim S. M.; Park D. J.; Park S. Surface Plasmon Resonance Extension Through Two-block Metal-conducting Polymer Nanorods. Nat. Commun. 2018, 9, 1010 10.1038/s41467-018-03453-z. 29520100 PMC5843636

[ref20] König T. A. F.; Ledin P. A.; Kerszulis J.; Mahmoud M. A.; El-Sayed M. A.; Reynolds J. R.; Tsukruk V. V. Electrically Tunable Plasmonic Behavior of Nanocube–Polymer Nanomaterials Induced by a Redox-Active Electrochromic Polymer. ACS Nano 2014, 8, 6182–6192. 10.1021/nn501601e. 24870253

[ref21] Lu W.; Jiang N.; Wang J. Active Electrochemical Plasmonic Switching on Polyaniline-Coated Gold Nanocrystals. Adv. Mater. 2017, 29, 1604862 10.1002/adma.201604862. 28004862

[ref22] Liang L.; Lam S. H.; Ma L.; Lu W.; Wang S.-B.; Chen A.; Wang J.; Shao L.; Jiang N. (Gold Nanorod Core)/(poly(3,4-ethylene-dioxythiophene) Shell) Nanostructures and Their Monolayer Arrays for Plasmonic Switching. Nanoscale 2020, 12, 20684–20692. 10.1039/D0NR05502F. 33047771

[ref23] Lin H.; Song L.; Huang Y.; Cheng Q.; Yang Y.; Guo Z.; Su F.; Chen T. Macroscopic Au@PANI Core/Shell Nanoparticle Superlattice Monolayer Film with Dual-Responsive Plasmonic Switches. ACS Appl. Mater. Interfaces 2020, 12, 11296–11304. 10.1021/acsami.0c01983. 32043861

[ref24] Lu W.; Chow T. H.; Lai S. N.; Zheng B.; Wang J. Electrochemical Switching of Plasmonic Colors Based on Polyaniline-coated Plasmonic Nanocrystals. ACS Appl. Mater. Interfaces 2020, 12, 17733–17744. 10.1021/acsami.0c01562. 32195574

[ref25] Peng J.; Jeong H.-H.; Lin Q.; Cormier S.; Liang H.-L.; Volder M. F. L. D.; Vignolini S.; Baumberg J. J. Scalable electrochromic Nanopixels Using Plasmonics. Sci. Adv. 2019, 5, eaaw2205 10.1126/sciadv.aaw2205. 31093530 PMC6510554

[ref26] Reiser B.; González-García L.; Kanelidis I.; Maurer J. H. M.; Kraus T. Gold Nanorods with Conjugated Polymer Ligands: Sintering-free Conductive Inks for Printed Electronics. Chem. Sci. 2016, 7, 4190–4196. 10.1039/C6SC00142D. 30155064 PMC6014069

[ref27] Contreras-Caceres R.; Alonso-Cristobal P.; Mendez-Gonzalez D.; Laurenti M.; Maldonado-Valdivia A.; Garcia-Blanco F.; López Cabarcos E.; Fernandez-Barbero A.; Lopez-Romero J. M.; Rubio-Retama J. Temperature Controlled Fluorescence on Au@Ag@PNIPAM-PTEBS Microgels: Effect of the Metal Core Size on the MEF Extension. Langmuir 2014, 30, 15560–15567. 10.1021/la503864f. 25437749

[ref28] Das S.; Chatterjee D. P.; Ghosh R.; Nandi A. K. Water soluble Polythiophenes: Preparation and Applications. RSC Adv. 2015, 5, 20160–20177. 10.1039/C4RA16496B.

[ref29] Yao Z.; Feng X.; Hong W.; Li C.; Shi G. A Simple Approach For the Discrimination of Nucleotides Based on a Water-soluble Polythiophene Derivative. Chem. Commun. 2009, 4696–4698. 10.1039/b904975d. 19641813

[ref30] Vial S.; Pastoriza-Santos I.; Pérez-Juste J.; Liz-Marzán L. M. Plasmon Coupling in Layer-by-Layer Assembled Gold Nanorod Films. Langmuir 2007, 23, 4606–4611. 10.1021/la063753t. 17367179

[ref31] Novo C.; Funston A. M.; Mulvaney P. Direct Observation of Chemical Reactions on Single Gold Nanocrystals Using Surface Plasmon Spectroscopy. Nat. Nanotechnol. 2008, 3, 598–602. 10.1038/nnano.2008.246. 18838998

[ref32] Novo C.; Funston A. M.; Gooding A. K.; Mulvaney P. Electrochemical Charging of Single Gold Nanorods. J. Am. Chem. Soc. 2009, 131, 14664–14666. 10.1021/ja905216h. 19824726

[ref33] Häkkinen H. The Gold–sulfur Interface at the Nanoscale. Nat. Chem. 2012, 4, 443–455. 10.1038/nchem.1352. 22614378

[ref34] Liu Y.-F.; Krug K.; Lee Y.-L. Self-organization of Two-dimensional Poly(3-hexylthiophene) Crystals on Au(111) Surfaces. Nanoscale 2013, 5, 7936–7941. 10.1039/c3nr02233a. 23857255

[ref35] Sahu D.; Chu H.-C.; Yang P.-J.; Lin H.-C. Surface Modification of Gold Nanorods by Grafting Fluorene-Based Conjugated Copolymers Containing Thiol-Pendants. Macromol. Chem. Phys. 2012, 213, 1550–1558. 10.1002/macp.201100550.

[ref36] Op de Beeck M.; Van Dyck D. Direct Structure Reconstruction in HRTEM. Ultramicroscopy 1996, 64, 153–165. 10.1016/0304-3991(96)00006-X.

[ref37] Choueiri R. M.; Galati E.; Thérien-Aubin H.; Klinkova A.; Larin E. M.; Querejeta-Fernández A.; Han L.; Xin H. L.; Gang O.; Zhulina E. B.; et al. Surface Patterning of Nanoparticles with Polymer Patches. Nature 2016, 538, 79–83. 10.1038/nature19089. 27556943 PMC5161688

[ref38] Backes I. K.; Gonzalez-Garcia L.; Holtsch A.; Muller F.; Jacobs K.; Kraus T. Molecular Origin of Electrical Conductivity in Gold–Polythiophene Hybrid Particle Films. J. Phys. Chem. Lett. 2020, 11, 10538–10547. 10.1021/acs.jpclett.0c02831. 33290078

[ref39] Britton J.; Majumdar S.; Weiss G. A. Continuous Flow Biocatalysis. Chem. Soc. Rev. 2018, 47, 5891–5918. 10.1039/C7CS00906B. 29922795 PMC6441135

[ref40] Mordhorst S.; Andexer J. N. Round, Round We Go – Strategies for Enzymatic Cofactor Regeneration. Nat. Prod. Rep. 2020, 37, 1316–1333. 10.1039/D0NP00004C. 32582886

[ref41] Zhang Y.; Zhao Y.; Li R.; Liu J. Bioinspired NADH Regeneration Based on Conjugated Photocatalytic Systems. Solar RRL 2021, 5, 2000339 10.1002/solr.202000339.

[ref42] Kinastowska K.; Liu J.; Tobin J. M.; Rakovich Y.; Vilela F.; Xu Z.; Bartkowiak W.; Grzelczak M. Photocatalytic Cofactor Regeneration Involving Triethanolamine Revisited: The Critical Role of Glycolaldehyde. Appl. Catal., B 2019, 243, 686–692. 10.1016/j.apcatb.2018.10.077.

[ref43] Sánchez-Iglesias A.; Chuvilin A.; Grzelczak M. Plasmon-driven Photoregeneration of Cofactor Molecules. ChemComm 2015, 51, 5330–5333. 10.1039/C4CC07829B. 25347548

[ref44] Sánchez-Iglesias A.; Barroso J.; Martinez-Solis D.; Taboada J. M.; Obelleiro-Basteiro F.; Pavlov V.; Chuvilin A.; Grzelczak M. Plasmonic Substrates Comprising Gold Nanostars Efficiently Regenerate Cofactor Molecules. J. Mater. Chem. A 2016, 4, 7045–7052. 10.1039/C6TA01770C.

[ref45] Kim Y.-S.; McNiven S.; Ikebukuro K.; Karube I. Continuous Photoreduction of Methyl Viologen Using Disubstituted Terthiophenes and EDTA in Aqueous Solution. Photochem. Photobiol. 1997, 66, 180–184. 10.1111/j.1751-1097.1997.tb08640.x.

[ref46] Minudri D.; Mantione D.; Dominguez-Alfaro A.; Moya S.; Maza E.; Bellacanzone C.; Antognazza M. R.; Mecerreyes D. Water Soluble Cationic Poly(3,4-Ethylenedioxythiophene) PEDOT-N as a Versatile Conducting Polymer for Bioelectronics. Adv. Electron. Mater. 2020, 6, 2000510 10.1002/aelm.202000510.

