# Supplementary material for: Metal–Polymer Heterojunction in Colloidal-Phase Plasmonic Catalysis
Source: J Phys Chem Lett. 2022 Mar 3;13(10):2264–72. doi: 10.1021/acs.jpclett.1c04242 (PMC8935371; doi:10.1021/acs.jpclett.1c04242)
Supplement: Supplementary file 1 — jz1c04242_si_001.pdf [file jz1c04242_si_001.pdf]

Supporting Information

Metal-polymer Heterojunction in Colloidal-phase Plasmonic Catalysis

Andrea Rogolino,[†] Nathalie Claes,[‡] Judit Cizaurre,[¶] Aimar Marauri,[¶] Alba Jumbo-Nogales,[§] Zuzanna Lawera,[§] Joscha Kruse,[§,∥] María Sanromán-Iglesias,[§] Ibai Zarketa,[¶] Unai Calvo,[¶] Elisa Jimenez-Izal,[¶,∥,⊥] Yury P. Rakovich,[§,∥,⊥] Sara Bals,[‡] Jon M. Matxain,[*,¶,∥] and Marek Grzelczak[*,§,∥]

†*Galilean School of Higher Education, University of Padova, Italy*

‡*EMAT-University of Antwerp, Groenenborgerlaan 171, B-2020 Antwerp, Belgium*

¶*Kimika Fakultatea, Euskal Herriko Unibertsitatea (UPV/EHU) Lardizabal Pasealekua 3, 20018, Donostia-San Sebastián, Spain*

§*Centro de Física de Materiales (CSIC-UPV/EHU), Paseo Manuel de Lardizabal 5, 20018 Donostia-Sebastián, Spain*

∥*Donostia International Physics Center (DIPC), Paseo Manuel de Lardizabal 4, 20018 Donostia-Sebastián, Spain*

⊥*Ikerbasque, Basque Foundation for Science, Bilbao, Spain*

E-mail: jonmattin.matxain@ehu.eus; marek.g@csic.es

Contents

1 Experimental Part **4**

1.1 Materials 4

1.2 Synthesis of gold nanospheres 4

1.3 Synthesis of gold nanocubes 5

1.4 Synthesis of gold nanorods 5

1.5 Synthesis of gold bipyramids 6

1.6 Ligand exchange of gold nanoparticles with PTEBS 6

1.7 Ligand exchange of gold nanoparticles with PSS 6

1.8 Ligand exchange of gold nanorods with MUS 7

1.9 Kinetics measurements of ligand exchange 7

1.10 PTEBS-TEAOH and PTEBS-NAD^+ interactions 7

1.11 NADH photoregeneration 8

1.12 Characterization 8

2 Characterization **9**

2.1 Exit Wave reconstruction of polymer shell 9

2.2 TEM characterization 10

2.3 DLS of spherical nanoparticles 11

3 NADH photoregeneration **12**

4 Control experiments **16**

5 Computational Simulations **17**

5.1 Computational details 17

5.1.1 Polymer structure 17

5.1.2 Metal-Polymer heterojunction 17

5.2 Models 18

5.2.1 Polymer structure 18
5.2.2 Metal-Polymer heterojunction 19

References **21**

1 Experimental Part

1.1 Materials

Sodium citrate, sodium chloride, tetrachloroauric (III) acid ($HAuCl_4$), hydrochloric acid, sodium borohydride ($NaBH_4$) cetyltrimethylammonium bromide (CTAB), cetyltrimethylammonium chloride (CTAC, 25 wt. % in water), benzyldimethylhexadecylammonium chloride (BDAC), sodium bromide, Polystyrene sulfonate (PSS, 70kg/mol), ascorbic acid (AA), citric acid, trisodium citrate, triethanolamine (TEAOH), β -Nicotinamide adenine dinucleotide sodium salt (NAD^+) and β -Nicotinamide adenine dinucleotide reduced disodium salt hydrate (NADH) were purchased from Merck Sigma-Aldrich. Poly[2-(3-thienyl)-ethyloxy-4-butylsulfonate] (PTEBS) was purchased by Solaris Chem. Sodium 11 - mercapto - 1 - undecanesulfonate (MUS) was purchased from ProChimia Surfaces. Milli-Q water was used in all the experiments. All glassware was cleaned with aqua regia and rinsed thoroughly with Milli-Q water.

1.2 Synthesis of gold nanospheres

To obtain spherical gold nanoparticles (Turkevich methods) an aqueous solution of trisodium citrate (150 mL, 2.2 mM) was heated for 15 min under stirring until boiling, followed by the injection of $HAuCl_4$ (1 mL, 25 mM). These nanoparticles were subjected to PTEBS coating. To obtain larger citrate-stabilized nanoparticles, the initial nanoparticles were overgrown by cyclic addition of $HAuCl_4$ (1 mL, 25 mM) at 90 °C in two steps. Then, a portion of growth solution (55 mL) was removed and the remaining solution (98 mL) was mixed with water (53 mL) and sodium citrate (2 mL, 60 mM). This process was repeated twice to obtain nanoparticles of $\sim$30 nm in diameter.

1.3 Synthesis of gold nanocubes

Gold nanocubes were prepared according to previously reported receipt.[1] Gold seeds nanoparticles were obtained through overgrowth of initial seeds nanoparticles of 1-2 nm. The initial seeds were prepared by reduction of $HAuCl_4$ (5 mL, 0.25 mM) with $NaBH_4$ (0.3 mL, 10 mM) in aqueous CTAB solution (100 mM). After complete decomposition of $NaBH_4$, the seed solution (0.11 mL) was added to a growth solution containing CTAC (20 mL, 200 mM), $HAuCl_4$ (20 mL, 0.5 mM) and AA (15 mL, 100mM). The mixture was left undisturbed for 30 min at 27 °C. To remove the excess of reagents, the mixture was centrifuged and redispersed in water to reach $[Au^0] \sim 3$ mM. The resulting solution of 10 nm gold nanospheres was used as seed in the second overgrowth. A solution of gold seeds (10 nm) (0.28 mL, 3 mM) was added at 40 °C to an aqueous solution containing BDAC (50 mL, 100 mM), $HAuCl_4$ (0.25 mL, 50 mM) and AA (0.25 mL, 100 mM). The mixture was left undisturbed at 30 °C for 30 min. The solution was centrifuged twice and redispersed in water to reach $[Au^0] \sim 7.5$ mM. To a solution containing CTAC (5mL, 15 mM) and overgrown Au seeds (0.12 mL, 7.5 mM) was added NaBr (0.5 mL, 10 mM), AA (0.0188 mL, 100 mM) and $HAuCl_4$ (0.025 mL, 50 mM). The mixtures were left under stirring for 30 min at room temperature followed by centrifugation.

1.4 Synthesis of gold nanorods

Gold nanorods were prepared using a Ag-assisted seeded growth method.[2] Seeds were prepared by reduction of $HAuCl_4$ (0.025 mL, 0.05 M) with $NaBH_4$ (0.3 mL, 0.01 M) in an aqueous CTAB solution (4.7 mL, 0.1 M) under vigorous stirring. The mixture was left undisturbed for 30 min. To the growth solution comprising CTAB (10 mL, 0.1 M) and $HAuCl_4$ (0.1 mL, 0.05 M) the following solutions were added: AA (0.08 mL, 0.1 M), HCl (0.19 mL, 1 M), $AgNO_3$ (0.12 mL, 0.01 M) and Au seed solution (0.024 mL). Between each addition, the vial was closed and vigorously shaken for at least 10 seconds. The mixture was left undisturbed at room temperature for at least 2 h. The final concentration of metallic

gold was $[Au^0] \sim 0.5$ mM.

1.5 Synthesis of gold bipyramids

Gold bipyramids were prepared using seeded growth method.[3] Seeds were prepared by fast injection of $NaBH_4$ (0.3 mL, 0.01 M) to a mixture containing $HAuCl_4$ (0.025 mL, 0.05 M), CTAC solution (4.7 mL, 0.05 M) and citric acid (1 M, 0.025 mL) under vigorous stirring. After assuring that all metallic gold was reduced (UV-Vis spectroscopy), the mixture was left undisturbed in water bath at 80 °C for 90 min. Gold bipyramids were prepared by adding an aliquot of gold seeds (1.1 mL) to a growth solution of CTAB (10 mL, 100 mM), $HAuCl_4$ (0.5 mL, 10 mM), $AgNO_3$ (0.1 mL, 10 mM), HCl (0.2 mL, 1 M) and AA (0.4 mL, 100 mM) at 30 °C. The mixture was left undisturbed at room temperature for at least 2 h.

1.6 Ligand exchange of gold nanoparticles with PTEBS

As-prepared solution of gold nanoparticles (40 mL, $[Au^0] \sim 0.5$ mM) was centrifuged and redispersed in water (40 mL) followed by the next centrifugation cycle again to be finally redispersed in water (20 mL). Such a solution contains surfactant of concentration close to critical micelle concentration. The nanoparticles solution (20 mL) was added to the PTEBS solution (20 mL, 0.3 mg/mL). The resulting mixture was left undisturbed for a day and subsequently centrifuged and redispersed in water to reach $[Au^0] \sim 1.3$ mM.

1.7 Ligand exchange of gold nanoparticles with PSS

A solution of gold nanorods (10 mL, $[Au^0] = 0.5$ mM, [CTAB] = 10 mM) was centrifuged and redispersed in an aqueous solution of PSS (10 mL, 0.15 wt%). The solution was centrifuged and redispersed in PSS (10 mL, 0.15 wt%).

1.8 Ligand exchange of gold nanorods with MUS

A solution of gold nanorods (10 mL, $[Au^0]$ = 0.5 mM, [CTAB] = 10 mM) was centrifuged and redispersed in water (8 mL). To this solution was added dropwise a solution of MUS (2 mL, 2.5 mg/mL) under vigorous stirring. The mixture was centrifuged and redispersed in water (10 mL).

1.9 Kinetics measurements of ligand exchange

A solution of PTEBS-coated gold nanorods (1 mL, $[Au^0] \sim 0.16$ mM) was placed in a plastic cuvette (optical path 1 cm, 4 mL) and left undisturbed for 5 minutes. The UV-Vis spectra were recorded in the interval of 1 minute. Then, a solution of PTEBS (2 mL) containing different polymer concentrations (0.10, 0.15, and 0.6 mg/mL) was injected. In control experiments, the same procedure was repeated replacing the polymer solution with water.

1.10 PTEBS-TEAOH and PTEBS-NAD^+ interactions

A stock solution of PTEBS (0.25 mg/mL) was prepared by dissolving the polymer in water 24 hours before the measurements and stirred at 40 °C. To study the effect of fluorescence emission in PTEBS-TEAOH system, ten samples were prepared which contained PTEBS (0.1 mg/mL) and TEAOH with increasing concentration in the following order: 0, 0.025, 0.050, 0.075, 0.100, 0.125, 0.150, 0.175, 0.200, 0.225, 0.250 M. Analogically, to study the effect of fluorescence emission in PTEBS-NAD^+ system, ten samples were prepared which contained PTEBS (0.1 mg/mL) and NAD^+ with increasing concentration in the following order: 0, 0.1, 0.2, 0.3, 0.4, 0.5, 0.6, 0.7, 0.8, 0.9, 1.0 mM. For all samples, absorbance and emission (excitation wavelength 390 nm) measurements were performed.

1.11 NADH photoregeneration

In a typical experiment, a quartz cuvette containing water (0.350 mL), TEAOH (0.5 mL, 3 M), NAD^+ (0.150 mL, 10 mM), Au@PTEBS (0.5 mL, $[Au^0] \sim 1.3$ mM) was placed in a four-windows cuvette holder coupled to a spectrophotometer and a light source in opposite windows and to two optical fibers from an halogen lamp in the remaining two opposite windows. The temperature of the cuvette holder was controlled by an external thermostat and recorded with a temperature logger equipped with thermocouple placed inside the cuvette. For each temperature (20, 35, 45, 50, 55 °C), the experiments were performed both under light and dark conditions. Under light conditions, the lamp (power density: 200 mW/cm^2) was turned on as soon as the cuvette was put in place. To acquire a UV-Vis spectrum that took ~20 s the lamp was turned off/on in 15 minutes interval.

1.12 Characterization

Time-dependent UV-vis-NIR measurements were performed on a Maya2000 Pro spectrophotometer (Ocean Optics) equipped with two optical fiber 400 μm in diameter and spectral range from 200 to 1100 nm. Temperature-dependent measurements of NADH oxidation were performed on a Cary 3500 equipped with thermal/kinetic module. Fluorescence emission spectra were acquired using Cary Eclipse. Fluorescence lifetime measurements were done using a MicroTime 200 confocal scanning lifetime fluorescent microscope (PicoQuant), completed with Olympus IX71 confocal microscope system and equipped with 485 nm ps diode laser and single photon avalanche diode detectors. X-ray photoelectron spectroscopy (XPS) measurements were performed using a SPECS Sage HR100 spectrometer equipped with a nonmonochromatic X-ray source (aluminum Kα line of 1486.6 eV, 300 W). Photocatalytic reactions were performed by using MI-150 halogen lamp by Dolan-Jenner Industries and Class AAA Pico LED solar simulator (G2V) of spectral range from 450 to 1200 nm under AM 1.5G filter. Temperature was measured with a Thorlabs TSP01 sensor. The hydrodynamic radius and Z-potential was acquired with a Zetasizer Nano ZS (Malvern analytical).

TEM images were collected with a JEOL (120 kV) microscope. Open source Python libraries (Pandas, Numpy, Scipy, Matplotlib) were used for data analysis and preparation of figures.

2 Characterization

2.1 Exit Wave reconstruction of polymer shell

A HRTEM image is a complex interference pattern formed by the interaction of the electron beam with the specimen and influenced by the electron optical system of the microscope. As a consequence, the interpretation of HRTEM images is far from intuitive, especially for non-periodic systems such as the polymer in this study. Therefore we used exit wave reconstruction, a technique based on the acquisition of a focal series of HRTEM images. By inverting the image formation process and hereby eliminating the lens aberrations, all information can be recovered up to the microscope's information limit. From the reconstructed exit wave, the phase image can be extracted, which makes direct interpretation of the atomic structure possible.[4] Indeed, when the sample is sufficiently thin, the reconstructed phase image is proportional to the projection potential, this means the projected atomic arrangement.

For High Resolution Transmission Electron Microscopy (HRTEM) characterisation an aliquot of sample solution (3 μL) was deposited on a graphene grid. The images were acquired using a monochromated aberration corrected Titan microscope (Thermo Fischer Scientific) operated at 80 kV using a negative spherical aberration (Cs=-5μm). Focal series for exit wave reconstruction consisted of 30 images acquired with a focal range of 15 nm around the focal point of the polymer and a focus increment of 0.5 nm. Reconstruction of the exit wave and offline correction of residual aberrations was carried out using the Gerchberg-Saxton algorithm implemented in the XWave software.

2.2 TEM characterization

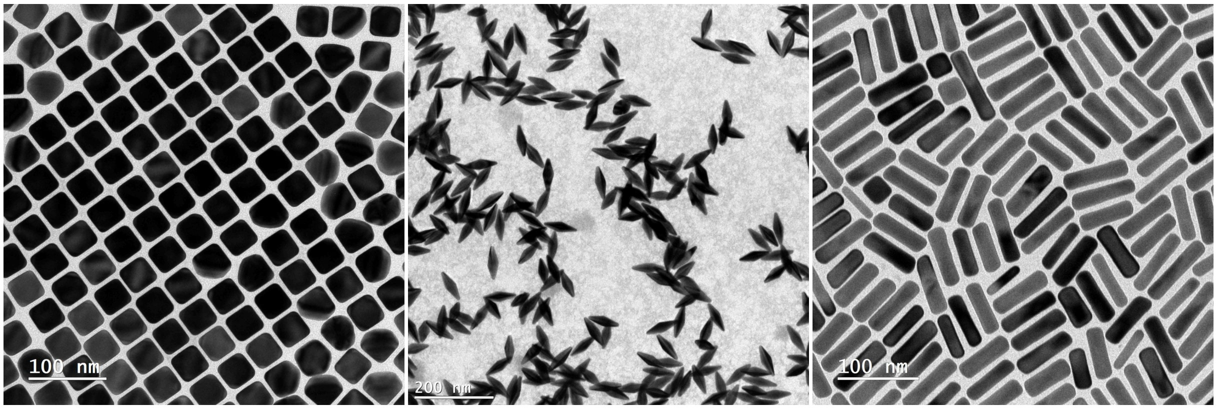

Figure S1: TEM images of initial nanocubes, bipyramids and nanorods

2.3 DLS of spherical nanoparticles

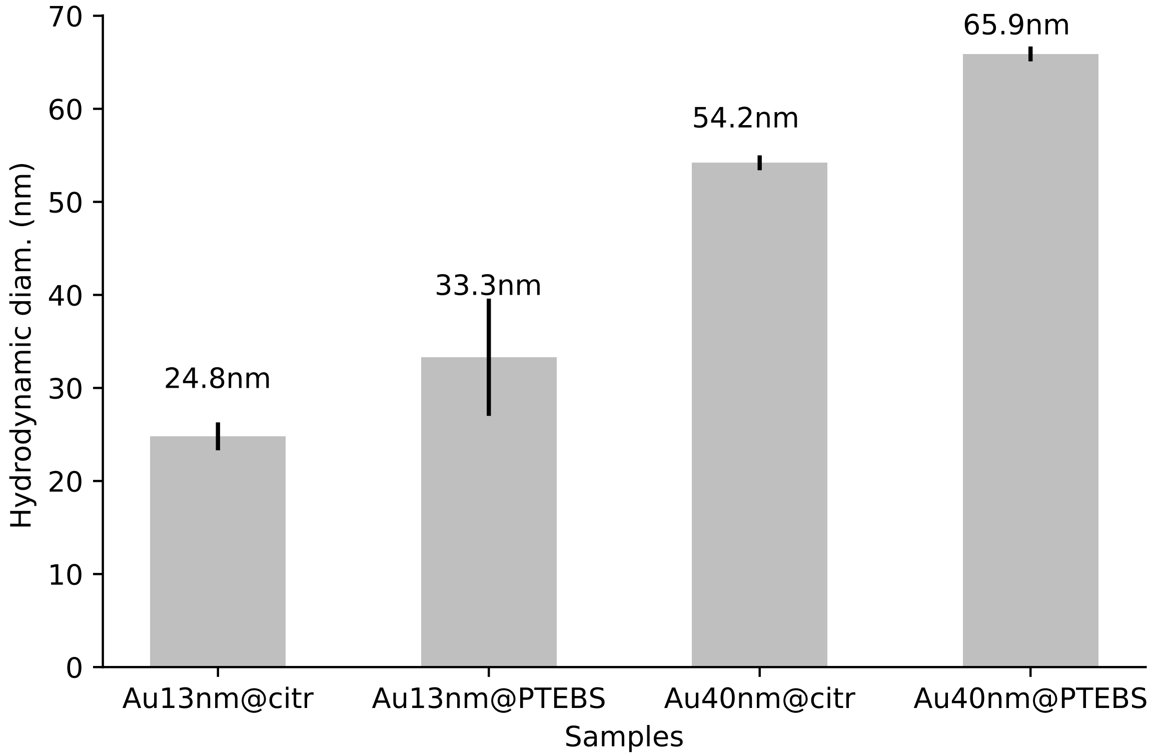

Figure S2: Hydrodynamic diameter of gold nanoparticles before and after ligand exchange. To show how hydrodynamic diameter increases after ligand exchange we used two diameters of nanoparticles, namely 13 and 40 nm. It is clear that the presence of PTEBS on nanoparticles surface increases the value of hydrodynamic diameter by ∼10 nm.

3 NADH photoregeneration

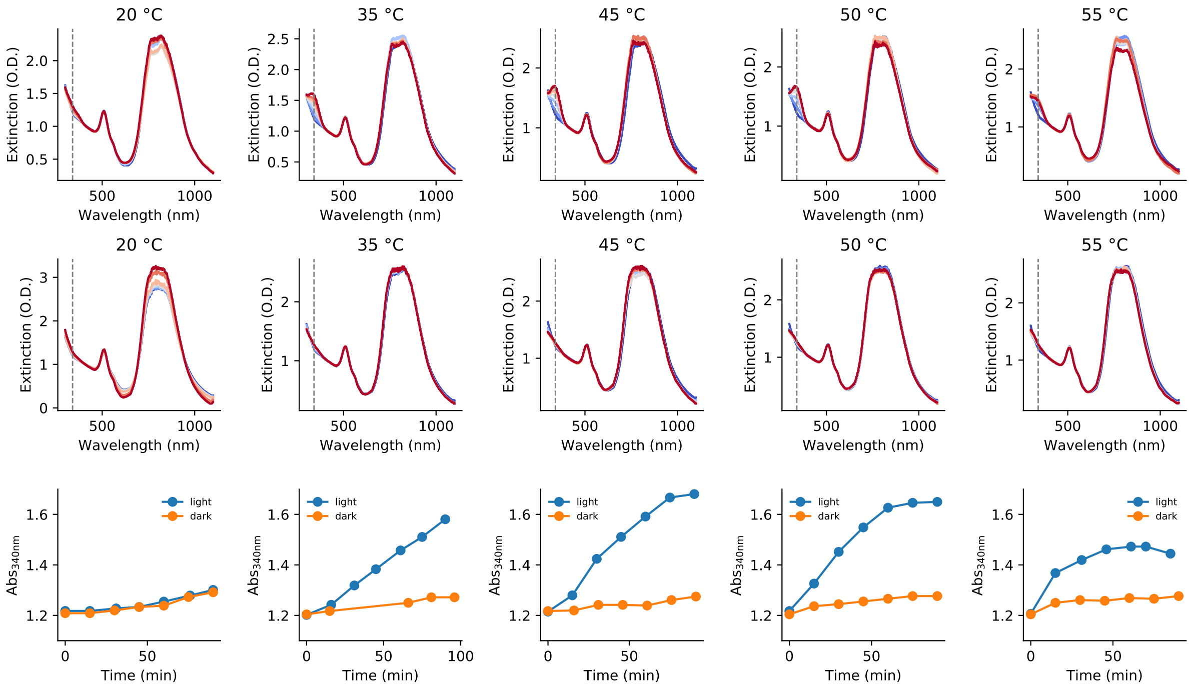

Figure S3: Regeneration of NADH on Au@PTEBS at different temperatures. (Upper panel) Light process. UV-vis-NIR spectra of mixture containing gold nanorods (Au=0.5 mM), $[NAD^+]$ = 1 mM, TEAOH = 1 M under light irradiation (450-1100nm, 200 mW/cm^2) for temperatures ranging from 20 to 55 °C. Each spectrum was taken every 5 minutes during 90 minutes of reaction. Note that the LSPR bands of gold nanorods are cutoff because of detector saturation. (Middle panel) Control in dark. The same experimental conditions as in upper panel, but in the absence of light, showing no NADH evolution. The dashed lines correspond to the absorbance of NADH (340 nm). (Lower panel) Time-depended change of absorbance at 340 nm for light and dark processes, showing that at 20 °C, no photo regeneration of NADH is observed.

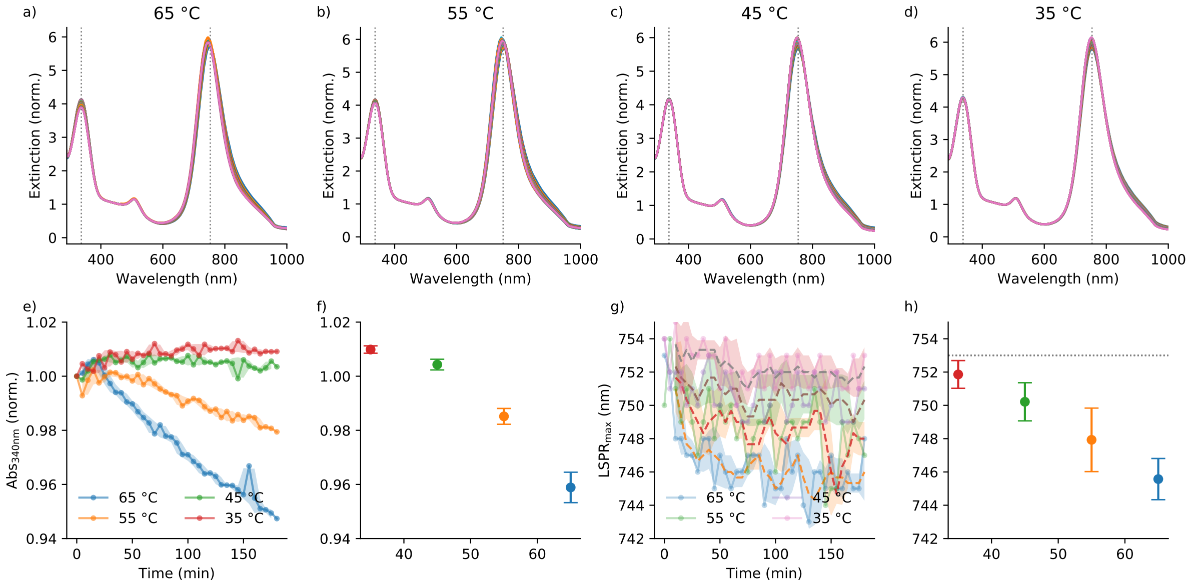

Figure S4: Effect of temperature on the oxidation of NADH in the presence of Au@PTEBS and TEAOH. (a-d) UV-vis-NIR spectra of mixtures at temperatures from 35 to 65 °C taken every 5 minutes for 3 hours. Experimental conditions : $[Au^0] = 0.125$ mM, $[NADH] = 0.1$ mM, $[TEAOH] = 1M$. e) Time-dependent absorption at 340 nm (dashed lines in a-d at 340 nm) for different temperatures, showing decreases of absorbance at 55 and 65 °C, suggesting oxidation of NADH to NAD^+ with increasing temperature. f) Absorbance at 340 nm, averaged over the last 15 points of the kinetics, versus temperature, showing non linear relation of NADH oxidation with change of temperature. Error bars for each point were obtained by calculating the standard deviation over the last 15 points of the kinetics. g) Time-dependent change of the maximum of LSPR for different temperatures, showing that the higher the temperature, the higher the blueshift of LSPR. h) position of the maxima of LSPR, averaged over the last 15 points of the kinetics, versus temperature, showing linear dependency of plasmon band blueshift with increasing temperature. The blueshift suggests the charging of the nanorods by oxidation of NADH to NAD^+

.

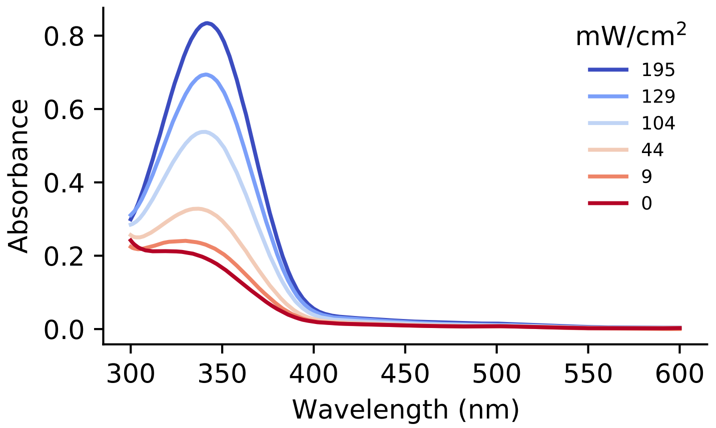

Figure S5: Effect of incident light power density on the photo regeneration of NADH using Au@PTEBS as photocatalyst. UV-vis-NIR spectra of centrifuged solutions, showing gradual increase of absorbance at 340 nm with increasing power density of incident light. Experimental conditions: [Au^0] = 0.5 mM, [NAD^+] = 1 mM, TEAOH = 1M, temperature = 35 °C, spectral range = 450-1200 nm.

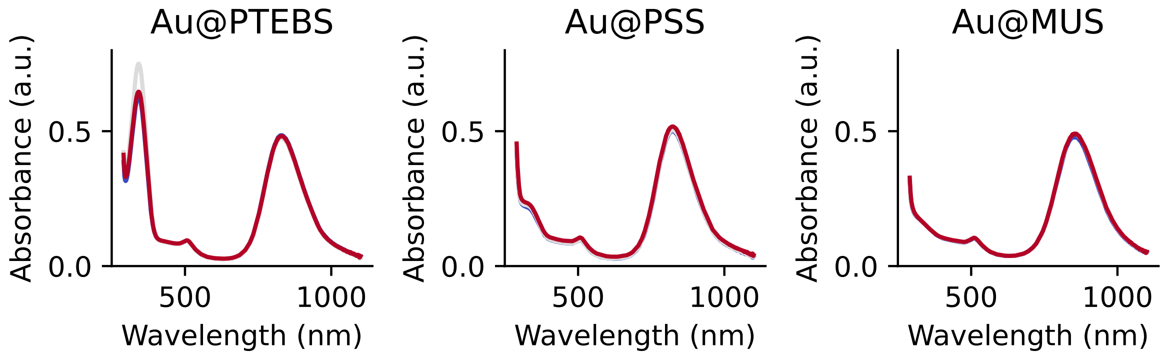

Figure S6: UV-vis-NIR spectra of photocatalytic mixtures containing gold nanorods functionalized with PTEBS (left), PSS (middle) and MUS (right). Experimental conditions: [Au^0] = 0.10 mM, [NAD^+] = 1 mM, TEAOH = 0.75 M, temperature = 35 °C, spectral range of incident light = 450-1200 nm, power density 120 mW/cm^2

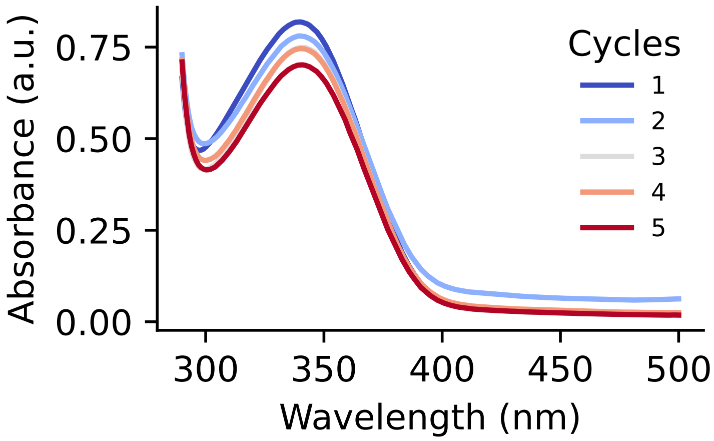

Figure S7: UV-vis spectra of centrifuged mixtures after each cycle of photocatalytic run. Experimental conditions: $[Au^0] = 0.10$ mM, $[NAD^+] = 1$ mM, TEAOH = 0.75 M, temperature = 35 °C, spectral range of incident light = 450-1200 nm, power density 120 mW/cm^2

4 Control experiments

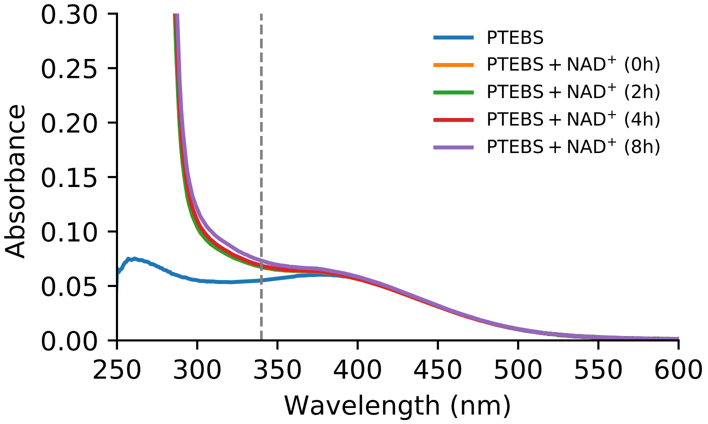

Figure S8: Control experiment showing that PTEBS alone is unable to reduce NAD^+ to NADH.

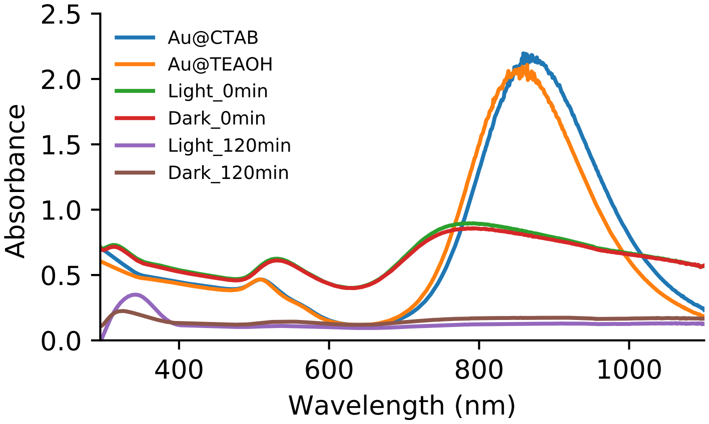

Figure S9: Control experiment. UV-vis-NIR spectra of CTAB-coated gold nanorods (blue) and after redispersion in TEAOH (orange). The initial stability of nanoparticles in TEAOH suggests that amine functional groups provide sufficient colloidal stability of nanoparticles, interaction of amine with gold surface, as predicted numerically (see main text). The addition of NAD^+ (1 mM) causes immediate aggregation (green, red). On contrary to the sample stored in dark (brown), the sample exposed to visible light (450-1200 nm, 200 mW/cm^2) for two hours revealed the presence of a band at 340 nm (violet). Both samples, however, aggregated, suggesting that the use of molecular interface - polymer - is crucial to preserve initial colloidal stability of the nanoparticles.

5 Computational Simulations

In order to study the metal-polymer hetero-junction, DFT simulations were performed on model systems. Before studying the metal-polymer interface, it is necessary to have some information regarding the polymer structure. In general, a polymer structure is complex and depends on many factors such as chain size and mobility. The simulation of all these features is extremely complex and it will be the subject of future studies. Instead, in this work we focused on finding the most stable crystal structure conformations for infinitely long polymer chains using Plane-Wave DFT (PW-DFT) along with Periodic Boundary Conditions. The methodology is described in subsection 5.1 and the system models used throughout this work are detailed in subsection 5.2.

5.1 Computational details

5.1.1 Polymer structure

Polymer structure simulations were performed using plane-wave DFT (PW-DFT), along with periodic boundary conditions, by means of the exchange-correlation PBE functional[5] and PAW pseudopotentials,[5] as implemented in the VASP package.[5–9] In order to correctly describe the dispersion interactions, the DFT-D3 scheme was used.[10] The energy convergence and the force convergence criteria are set to be 10-5 eV and 0.05 eV/A, respectively. The plane wave cutoff energy was 400 eV, and a 3x9x21 Γ-centered k-point grid was used in the calculations.

5.1.2 Metal-Polymer heterojunction

To study the metal - polymer heterojunction, the Gaussian16 program package[11] was used to perform all the calculations. All geometry optimizations and harmonic frequency calculations were performed considering the solvent effect (water) by means of the integral equation formalism of the polarized continuum model (IEFPCM),[12] using the DFT formalism.[13,14] First,

geometry optimizations were performed by using the PBE exchange-correlation functional,[5] combined with the 6-31+G(d,p) basis set for the nonmetal atoms[15,16] and the LanL2DZ effective core potentials plus DZ basis set for gold atoms[17–19] including the D3 version of Grimme's dispersion with Becke-Johnson damping.[20] For optimized geometries, harmonic vibrational frequencies were computed by analytical differentiation of gradients, at the same level of theory, to ensure that the characterized structures were the true minima. Such frequencies were then used to evaluate the zero-point vibrational energy (ZPVE) and the thermal (T= 298 K) vibrational corrections to the enthalpy (H^{corr}). Then, single-point calculations using the 6-311++G(2df,2p) basis set[21] were performed on the optimized structures to refine the electronic energy (E_{elec}). In this way, the total enthalpies (H) of each species in solution were calculated as follows:

$$H = E_{elec} + H^{corr} \tag{1}$$

Finally, these enthalpy values were used to calculate the interaction enthalpies ΔH.

5.2 Models

5.2.1 Polymer structure

In order to determine the most stable structures of the polymer, two different structures within the unit cell were considered. Namely, one in which the dimer is in the cis conformation and another one with the dimer in the trans conformations of the polymeric chain, as depicted in Figure S10a. Based on these two unit cells, twenty starting geometries were generated in total, 10 for each conformation, by using different translational vectors. First, the polymeric chains were translated in one dimension and the thermodynamic stability was evaluated. The most stable structures correspond to the polymeric chain in the trans conformation, where the thiophene rings are coplanar (see Figure S10b). These interactions lead to strong π delocalisation that stabilises the structure. The thiophene rings are located

in the inner part of the chain, whereas the sulfonate groups are located in the outer part. Notice that up to three water molecules have been added in each sulfonate group, in order to balance the electrostatic interaction between this group and Na cations in solution. The polymeric structure is then grown in the other two directions with sulfonate - sulfonate interactions between chains, and π stacking between the aromatic rings. All these interactions are favorable, and lead to a family of stable crystal structures. The two most stable conformations are depicted in Figure S10c. Notice that the effect of the temperature is not considered in our calculations.

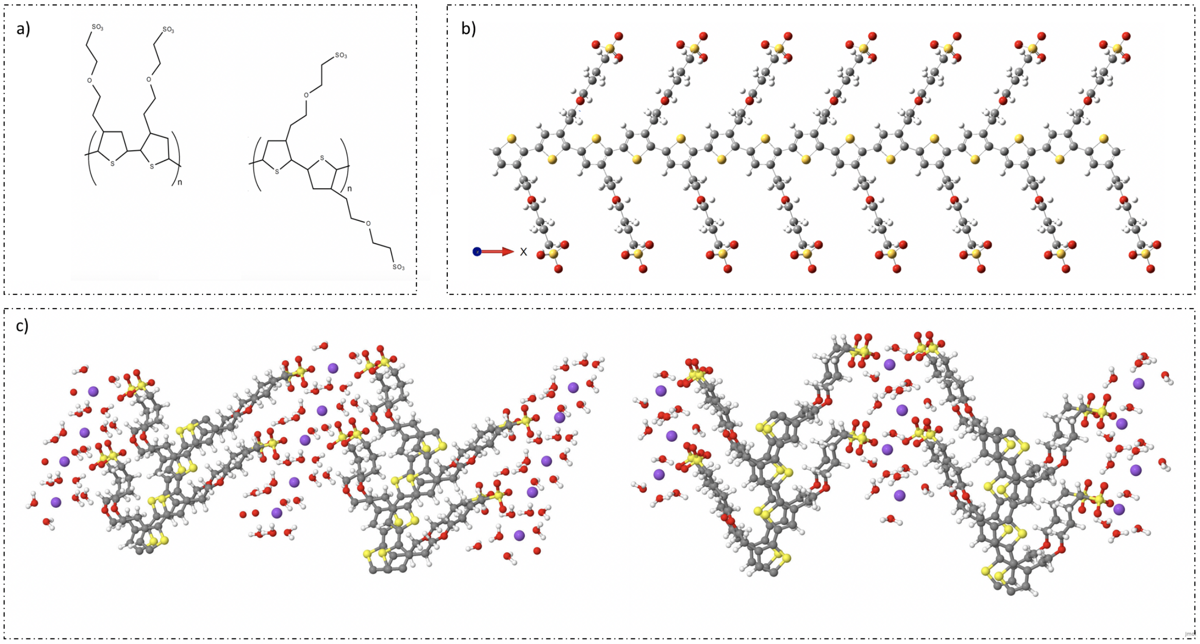

Figure S10: a) The two considered unit cells, cis and trans. b) Most stable infinitely long polymeric chain in trans conformation. Notice that in both a) and b) the added three water molecules and Na cations per sulfonate group are not depicted for the sake of clarity. c) Different conformations of the most stable polymeric crystal structures.

5.2.2 Metal-Polymer heterojunction

To study the metal-polymer heterojunction by means of DFT, we used simplified models shown in Figure S11. For the polymer different approaches were employed. On the one hand, basic constituents were used, as depicted in Figure S11a I, namely, methyl thiophene

dimers in cis and trans conformations and methyl sulfonate, including three water molecules as done in the polymer crystal simulations.

On the other hand, full TEBS dimers were considered, in both cis and trans conformations, analogously to the unit cells used in the polymer crystal simulations (see Figure S11a II). Gold nanoparticles were simulated using Au_{34} nanoclusters, depicted in Figure S11a III. Finally, triethanolamine species were modeled by adding three extra water molecules to each alcohol functional group, as shown in Figure S11a IV. The triethanolamine species may also interact with gold, and the calculated stable structure is depicted in Figure S11b.

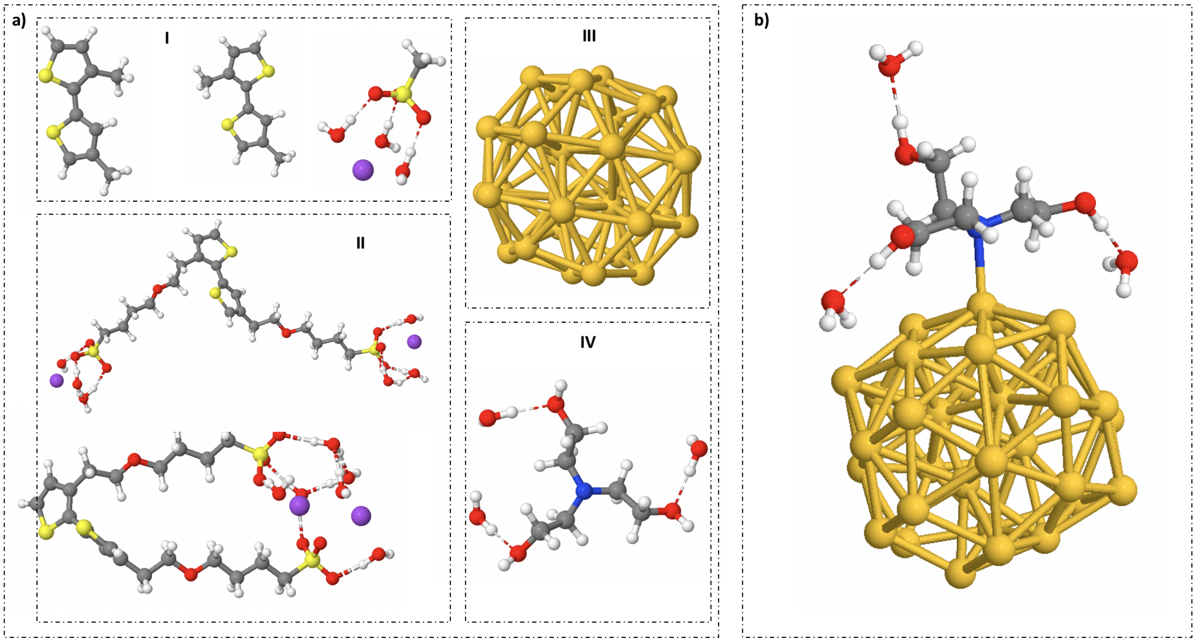

Figure S11: Models used for gold nanoparticles, PTEBS polymer and triethanolamine. a) Small polymer models (I), TEBS dimers in cis and trans conformations (II), Au_{34} nanocluster (III) and triethanolamine (IV). b) Gold - Triethanolamine interaction.

References

(1) Mezzasalma, S. A.; Grzelczak, M.; Sancho-Parramon, J. The Crystal Field Plasmon Splitting. *ACS Photonics* **2020**, *7*, 1551–1559.

(2) Liu, M.; Guyot-Sionnest, P. Mechanism of Silver(I)-Assisted Growth of Gold Nanorods and Bipyramids. *J. Phys. Chem. B* **2005**, *109*, 22192–22200.

(3) Sánchez-Iglesias, A.; Winckelmans, N.; Altantzis, T.; Bals, S.; Grzelczak, M.; Liz-Marzán, L. M. High-Yield Seeded Growth of Monodisperse Pentatwinned Gold Nanoparticles through Thermally Induced Seed Twinning. *J. Am. Chem. Soc.* **2017**, *139*, 107–110.

(4) Op de Beeck, M.; Van Dyck, D. Direct Structure Reconstruction in HRTEM. *Ultramicroscopy* **1996**, *64*, 153–165.

(5) Perdew, K., J. P.; Burke; Ernzerhof, M. Generalized Gradient Approximation Made Simple. *Phys. Rev. Lett.* **1996**, *77*, 3865.

(6) Baumann, T. F.; Gash, A. E.; Chinn, S. C.; Sawvel, A. M.; Maxwell, J. H., R. S.; Satcher Synthesis of High-Surface-Area Alumina Aerogels without the Use of Alkoxide Precursors. *Chem. Mater.* **2005**, *17*, 395.

(7) Kim, W.; Suh, D. J.; Park, T.; Hong, I. Photocatalytic degradation of methanol on titania and titania–silica aerogels prepared by non-alkoxide sol–gel route. *Top. Catal.* **2007**, *44*, 499.

(8) Kresse, G.; Furthmüller, J. Efficiency of ab-initio total energy calculations for metals and semiconductors using a plane-wave basis set. *Comput. Mater. Sci.* **1996**, *6*, 15.

(9) Kresse, G.; Furthmüller, J. Efficient iterative schemes for ab initio total-energy calculations using a plane-wave basis set. *Phys. Rev. B: Condens. Matter* **1996**, *54*, 11169.

(10) Grimme, S.; Antony, J.; Ehrlich, S.; ; Krieg, H. A consistent and accurate ab initio parametrization of density functional dispersion correction (DFT-D) for the 94 elements H-Pu. *J. Chem. Phys.* **2010**, *132*, 154184.

(11) Frisch, M. J. et al. . *Gaussian 16 Rev B.01; Wellingford, CT* **2016**,

(12) Scalmani, G.; Frisch, M. J. Continuous Surface ChargePolarizable Continuum Models of Solvation. I. General Formalism. *J. Chem. Phys.* **2010**, *132*, 114110.

(13) Hohenberg, P.; Kohn, W. Inhomogeneous Electron Gas. *Phys. Rev.* **1964**, *136*, B864–B871.

(14) Kohn, W.; Sham, L. J. Self-Consistent Equations IncludingExchange and Correlation Effects. *Phys. Rev.* **1965**, *140*, A1133–A1138.

(15) Hariharan, P. C.; Pople, J. A. Influence of Polarization Functions on Molecular-orbital Hydrogenation Energies. *Theor. Chem. Acc.* **1973**, *28*, 213–222.

(16) Franck, M. M.; Pietro, W. J.; Hehre, J. S.; Binkley, D. J.; DeFrees, J. A.; Pople, J. A.; Gordon, M. S. Self-consistent molecular orbital methods. XXIII. A polarization-type basis set for second-row elements. *J. Chem. Phys.* **1982**, *77*, 3654–3665.

(17) Hay, P. J.; Wadt, W. R. Ab initio effective core potentials for molecular calculations – potentials for the transition-metal atoms Sc to Hg. *J. Chem. Phys.* **1985**, *82*, 270.

(18) Wadt, W. R.; Hay, P. J. Ab initio effective core potentials for molecular calculations – potentials for main group elements Na to Bi. *J. Chem. Phys.* **1985**, *82*, 284.

(19) Hay, P. J.; Wadt, W. R. Ab initio effective core potentials for molecular calculations – potentials for K to Au including the outermost core orbitals. *J. Chem. Phys.* **1985**, *82*, 299.

(20) Grimme, S., S.; Ehrlich; Goerigk, L. Effect of the damping function in dispersion corrected density functional theory. *J. Comp. Phys.* **1456**, *32*, 1456.

(21) Krishnan, R.; Binkley, J. S.; Seeger, R.; Pople, J. A. Self-consistent Molecular Orbital Methods XX. A Basis Set for Correlated Wave Functions. *J. Chem. Phys.* **1980**, *72*, 650.
