# Supplementary material for: Metal–Polymer Heterojunction in Colloidal-Phase Plasmonic Catalysis
Source: J Phys Chem Lett. 2022 Mar 3;13(10):2264–72. doi: 10.1021/acs.jpclett.1c04242 (PMC8935371; doi:10.1021/acs.jpclett.1c04242)
Supplement: Supplementary file 2 — jz1c04242_si_002.pdf [file jz1c04242_si_002.pdf]

jz-2021-04242z.R1

Name: Peer Review Information for "Metal-Polymer Heterojunction in Colloidal-Phase Plasmonic Catalysis"

First Round of Reviewer Comments

Reviewer: 1

Comments to the Author

Journal: The Journal for Physical Chemistry Letters

Authors: Andrea Rogolino, Marek Grzelczak, and colleagues

Title: Metal-Polymer Heterojunction in Colloidal-Phase Plasmonic Catalysis

Review Report:

1. What is the major advance reported in the paper?

The authors prepared hybrid particles containing a gold core (with different geometries) covered with a shell of a water-soluble, conjugated polythiophene and studied its photocatalytical properties. They provide a detailed analysis of the ligand's binding to the metal surface. This careful analysis goes beyond previous reports on similar nanostructures and is useful to the community on its own. It could be further improved by linking DFT results and experimental observations on the electronic situation more closely (see "Technical suggestions" in section 3.)

The most important set of results, in my opinion, are those on the photocatalytic reduction of NAD^+ to NADH. The authors use the (non-Arrhenius) temperature dependence of the kinetics to support their claim of a photocatalytical mechanism and carefully consider photothermal hearing as an alternative explanation. I am missing reference experiments (see section 3) and I found the discussion on 11-13 somewhat meandering. Both can be ameliorated in a suitable revision, and if the resulting revised manuscripts demonstrated that the conjugated polymer acts both as a colloidal stabilizer and an electronic interface (or co-catalyst?) in the reaction, this is a major advance in my opinion.

2. What is the immediate significance of this advance?

The improved analysis of the gold-polythiophene hybrids supports previous reports and provides a clearer picture of the electronic situation in such constructs.

Most importantly, the work shows that the polythiophenes are stable enough at least for some photocatalytic reactions. It can and should show (see my comments below) that the electron transport through the organic ligand shell affects the catalytic process. This would, as the authors correctly point out, open a very attractive field of hybrid catalytic systems that use organic shells (that can be tuned in great detail electronically) to interact with substrates.

3. Technical suggestions

The authors provide an interesting discussion on the observed blueshift upon PTEBS adsorption on the gold surface. The key section (on page 5) of the discussion is very brief, and I do not understand which mechanism the authors see as connection between the polymer concentration in solution and the extend of blueshift. If (as the authors seem to claim) there are gold-sulfur bonds formed and if these are critical in the electronic situation, there should only be a difference if the concentration of polymer was so low that the particles' surfaces were not fully covered in some cases, and one would expect saturation once the concentration is sufficient to cover all. A somewhat more detailed discussion that considers the results of the DFT calculations presented on pages 7ff would make the manuscript stronger in my opinion.

I was surprised that the key experiments on photocatalysis (page 9ff, Figure 4) do not show results from reference materials, e.g. the same gold cores but with non-conjugated, polar organic shells (for example, self-assembled monolayers). The absolute minimum in my opinion would be a summary of related results from literature, but for the sake of comparison it would be very useful to see experimental results on non-conjugated shells. I suspect that the original ligand shell of the gold particles (CTAB) does not sufficiently stabilize the dispersion to perform such experiments, but commercial thiols exist that can do the job.

The authors should be lauded for discussing possible photothermal heating that may lead to non-Arrhenius kinetics and provide a possible alternative explanation for the observed catalytic effect. The discussion on page 11ff is detailed but loses, in my opinion, the connection to the original question. How likely is it that thermal effects (rather than plasmonic catalysis) are dominating the catalysis? A pointed conclusion would make the manuscript more readable and possible more convincing. If the authors do not want to take a clear stance, they should simply write this (they already do point out possibly experimental routes to a more detailed answer.)

Typo: "nm" is missing after 340 on page 10

Reviewer: 2

Comments to the Author

In this work, Rogolino et al report a water-soluble metal-polymer heterojunction (Au@PTEBS) in colloidal-phase, which is used in plasmonic catalysis for NADH regeneration. The in-situ spectroscopy, ex-situ advanced electron microscopy, molecular dynamics, and DFT calculations, et al., have been used to reveal the relationship between the polymer molecules and the metal surface. Furthermore, the photocatalytic activity of Au@PTEBS was tested in the light-assisted photoreduction of NAD+ to NADH using triethanolamine (TEAOH) as an electron donor. The work is solid and meaningful and therefore it can be considered for publication in prestigious The Journal of Physical Chemistry Letters after addressing the next remarks:

1. There are a lot of contents discussed about the material of the metal-polymer heterojunction in the “Abstract and Conclusion” section, but which is seldom focus on the NADH regeneration performance study. Please added the relative discussion.

2. Figures in the manuscript should be appeared in the normal order. For example, Figure 3d should be discussed after Figure 3a. Please adjust the sequence of the Figure or the modify the relative contents.

3. Please discussed the stability of the metal-polymer heterojunction in the manuscript. Such as the NADH regeneration experiments on cyclic stability tests should be added.

4. All Journal names should be abbreviated and unified in the References part. Some Journal names have been wrong, please corrected them.

5. Please supply the full English name of PEDOT in the manuscript.

6. Please modify the graphical TOC and supply a beautiful picture.

7. Hydrophilic and hydrophobic of the polymer plays an important role in the metal surface as well as the catalysis, please discussed it in detail in the manuscript.

Author's Response to Peer Review Comments:

Marek Grzelczak
CSIC Research Scientist
Center for Materials Physics CSIC-UPV/EHU
Manuel Lardizabal Ibilbidea 5,
20018 Donostia - San Sebastian
Phone: +34 943 018971
Email: marek.g@csic.es

February 19, 2022

Prof. Editor
Senior Editor
The Journal of Physical Chemistry Letters

Manuscript ID: jz-2021-04242z

Dear Dr. Editor,

Thank you for reviewing our manuscript titled "*Metal-Polymer Heterojunction in Colloidal-Phase Plasmonic Catalysis*", which we submitted to *The Journal of Physical Chemistry Letters*.

The reviews were mostly positive suggesting valuable changes, in particular Reviewer #1. We have addressed all remarks with corresponding edits to the manuscript and supporting information. Per your request, we have uploaded a revised version of our manuscript and supporting information, along with our point-by-point responses to the reviewers' comments.

Should you require any additional information, please let me know at your earliest convenience.

Sincerely,

Grzelczak

Marek Grzelczak
CSIC Research Scientist

Marek Grzelczak
CSIC Research Scientist
Center for Materials Physics CSIC-UPV/EHU
Manuel Lardizabal Ibilbidea 5,
20018 Donostia - San Sebastian
Phone: +34 943 018971
Email: marek.g@csic.es

A point-by-point response to reviewers' concerns:

REVIEWER # 1:

The authors prepared hybrid particles containing a gold core (with different geometries) covered with a shell of a water-soluble, conjugated polythiophene and studied its photocatalytical properties. They provide a detailed analysis of the ligand's binding to the metal surface. This careful analysis goes beyond previous reports on similar nanostructures and is useful to the community on its own. It could be further improved by linking DFT results and experimental observations on the electronic situation more closely (see "Technical suggestions" in section 3.) [see Question #2]

The most important set of results, in my opinion, are those on the photocatalytic reduction of NAD^+ to NADH. The authors use the (non-Arrhenius) temperature dependence of the kinetics to support their claim of a photocatalytical mechanism and carefully consider photothermal hearing as an alternative explanation. I am missing reference experiments (see section 3, Question #3) and I found the discussion on 11-13 somewhat meandering. Both can be ameliorated in a suitable revision, and if the resulting revised manuscripts demonstrated that the conjugated polymer acts both as a colloidal stabilizer and an electronic interface (or co-catalyst?) in the reaction, this is a major advance in my opinion.

The improved analysis of the gold-polythiophene hybrids supports previous reports and provides a clearer picture of the electronic situation in such constructs.

Most importantly, the work shows that the polythiophenes are stable enough at least for some photocatalytic reactions. It can and should show (see my comments below) that the electron transport through the organic ligand shell affects the catalytic process. This would, as the authors correctly point out, open a very attractive field of hybrid catalytic systems that use organic shells (that can be tuned in great detail electronically) to interact with substrates.

1. *The authors provide an interesting discussion on the observed blueshift upon PTEBS adsorption on the gold surface. The key section (on page 5) of the discussion is very brief, and I do not understand which mechanism the authors see as connection between the polymer concentration in solution and the extend of blueshift. If (as the authors seem to claim) there are gold-sulfur bonds formed and if these are critical in the electronic situation, there should only be a difference if the concentration of polymer was so low that the particles' surfaces were not fully covered in some cases, and one would expect saturation once the concentration is sufficient to cover all.*

This is an important question. To study the effect of polymer concentration on the blueshift of localized surface plasmon band (Figure 2e) we first needed to assure the colloidal stability of the nanoparticles during the process of ligand exchange. Below a concentration threshold (~5000 molecules per particle) we observed the aggregation of the nanoparticles due to the insufficient amount of polymer to cover the available metal surface. The reviewer is right by saying that electronic saturation should be strictly related to the physical saturation of the

Marek Grzelczak
CSIC Research Scientist
Center for Materials Physics CSIC-UPV/EHU
Manuel Lardizabal Ibilbidea 5,
20018 Donostia - San Sebastian
Phone: +34 943 018971
Email: marek.g@csic.es

metal surface, as also observed recently (e.g., DOI:10.1126/sciadv.aav0704). However, the detection of such saturation in the colloidal phase is unachievable experimentally because of a blueshift from the change of electronic structure can be altered by the shift (redshift or blueshift) due to aggregation. Therefore, we performed our experiments at the polymer concentration range above the aggregation threshold.

Nevertheless, we estimated that 70000 polymer molecules per particle correspond to 16 molecules per nm^2 of gold metal. After the formation of the first monolayer, there is also a possibility for electron transfer from the free polymer molecules in the solution, especially if assuming that the molecular shell is conductive. Therefore, in the present scenario, the electronic saturation of the plasmonic core is not strictly limited to the physical saturation of the surface by polymer, but it is rather related to the electronic properties polymeric shell.

To clarify this point, we have added the following sentences into the paragraph on Page 6:

> "Such an electron transfer can continue even after the formation of a compact polymeric shell, especially if there is an excess of free polymer in solution (~16 molecules per nm^2 of metallic gold) and assuming a conductive character of the polymer shell.[26] "

2. *A somewhat more detailed discussion that considers the results of the DFT calculations presented on pages 7 would make the manuscript stronger in my opinion.*

We thank the reviewer for this meaningful comment. Taking into account limited space in a format letter, we provided a comprehensive discussion of the DFT results in the supporting information file. By following the reviewer's suggestion, we have expanded the discussion related to the DFT on pages 8 and 9:

Last sentence in the first paragraph on Page 8:

> "The calculations confirmed that the polymer interacts with gold via sulphur atoms of thiophene rings, and oxygen atoms of the sulfonate group (Figure 3e, Table 1), suggesting that cooperative interactions ensure a homogeneous coverage of the gold nanoparticle."

Last sentence in the second paragraph on Page 8:

> "Nevertheless, the Au-S interatomic distances impose that only one of the sulfur atoms interacts with the nanocluster, conserving thus the ring coplanarity and the π delocalisation (the values of dihedral angle are close to 0 or 180 °)."

Third sentence in the last paragraph on Page 8:

> "Indeed, the C-S-S-C dihedral angle (related to the planarity between thiophene rings) in the cis conformation is enlarged from 2.5 to 17.8 degrees, indicating the breaking of

Marek Grzelczak
CSIC Research Scientist
Center for Materials Physics CSIC-UPV/EHU
Manuel Lardizabal Ibilbidea 5,
20018 Donostia - San Sebastian
Phone: +34 943 018971
Email: marek.g@csic.es

the π delocalization over the rings. This is not the case in the trans conformation, which conserves the ring planarity even in the PTEBS dimer model. These results indicate that not all sulphur atoms are capable of binding at the same time to the gold surface, collaborating the information obtained from HRTEM imaging. “

3. *I was surprised that the key experiments on photocatalysis (page 9, Figure 4) do not show results from reference materials, e.g. the same gold cores but with non-conjugated, polar organic shells (for example, self-assembled monolayers). The absolute minimum in my opinion would be a summary of related results from literature, but for the sake of comparison it would be very useful to see experimental results on non-conjugated shells. I suspect that the original ligand shell of the gold particles (CTAB) does not sufficiently stabilize the dispersion to perform such experiments, but commercial thiols exist that can do the job.*

We thank the reviewer for this meaningful comment. Indeed, the native surfactant (CTAB) is not a suitable molecular interface for a control experiment since the particles aggregate in the photocatalytic mixture. We followed the reviewer’s suggestion and performed additional experiments in which we compared PTEBS polymer with other non-conjugated molecular systems that comprise sulfonate and/or sulfphur. From a limited number of commercially available molecules, we selected the following molecular interface: polystyrene sulfonate (PSS) that contains sulfonate functional groups, and 11-mercapto-1-undecanesulfonate (MUS), an alkanethiol with a sulfonate functional group.
The three systems were subjected to a photocatalytic regeneration of NAD^+ to NADH, showing that gold nanorods coated with PTEBS outperform the gold nanorods coated with non-conjugated molecular shell. As pointed out by the reviewer, these results indicate that conjugated surface ligand allows for efficient metal-polymer heterojunction in plasmonic catalysis.

We have extended Figure 4 by adding a new subplot (Figure 4f):

Marek Grzelczak
CSIC Research Scientist
Center for Materials Physics CSIC-UPV/EHU
Manuel Lardizabal Ibilbidea 5,
20018 Donostia - San Sebastian
Phone: +34 943 018971
Email: marek.g@csic.es

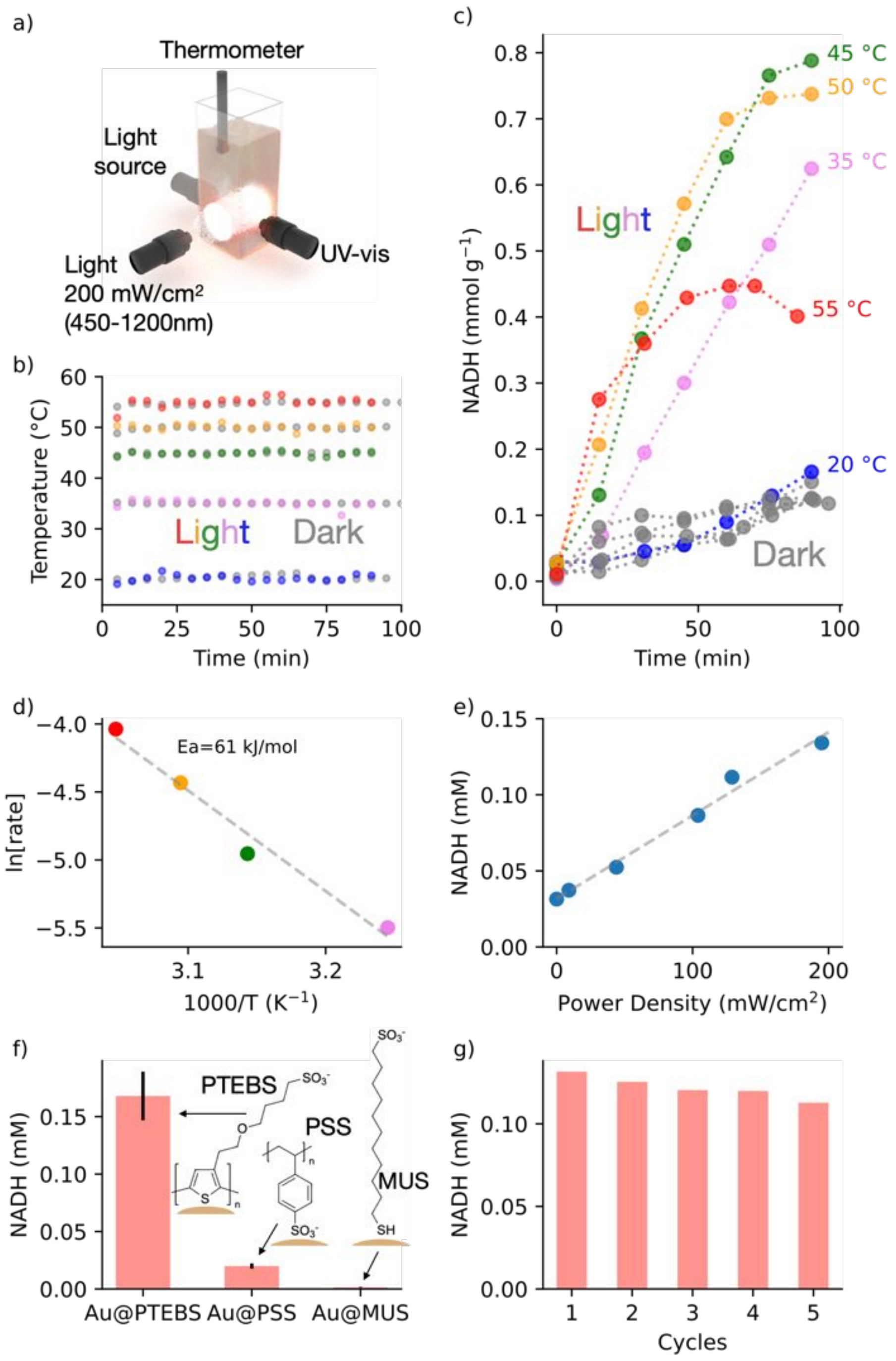

Figure 4. Photocatalytic regeneration of cofactor molecules on Au@PTEBS. a) Scheme of the reactor comprising real-time analytics: temperature and spectroscopy. b) Time-dependent evolution of NADH at temperatures ranging from 20 to 55 °C under light (coloured) and dark (grey) conditions. c) Steady-state temperature profile under light (coloured) and dark (grey) conditions. d) Arrhenius analysis of the process. e) Regeneration of NADH as a function of power density. f) Effect of surface ligand on NADH regeneration. g) Cyclic regeneration of NADH.

Marek Grzelczak
CSIC Research Scientist
Center for Materials Physics CSIC-UPV/EHU
Manuel Lardizabal Ibilbidea 5,
20018 Donostia - San Sebastian
Phone: +34 943 018971
Email: marek.g@csic.es

We have edited the paragraph on page 11 by providing the discussion on the new data:

> “To show the advantage of using a conjugated molecular shell we compared the photocatalytic performance of gold nanorods coated with other non-conjugated molecular systems. We selected a polymer comprising sulfonate groups, namely polystyrene sulfonate (PSS) and alkanethiol 11-mercapto-1-undecanesulfonate (MUS) that comprises both thiol and sulfonate functional groups. We observed that the system comprising conjugated polymer outperforms PSS and MUS (Figure 4f and Figure S6), confirming that conjugated molecular shell in metal-polymer heterojunction allows for efficient electron transfer in redox reactions.”

In addition, we have introduced a new figure in the supporting information (Page 14):

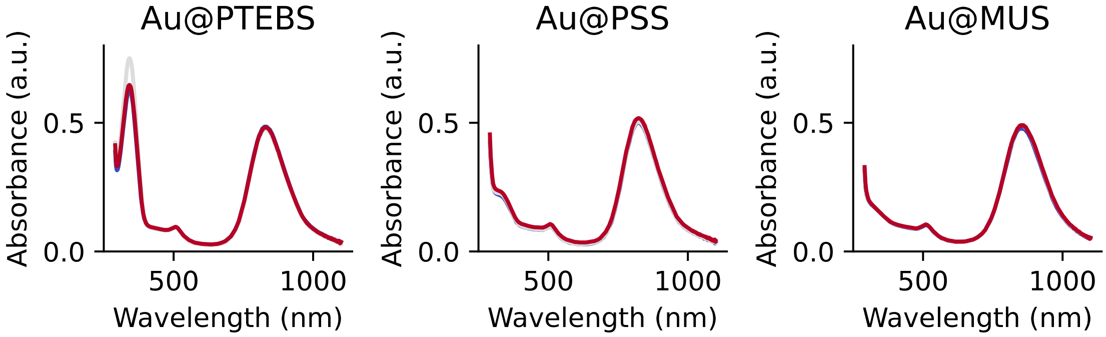

Figure S6. UV-vis-NIR spectra of photocatalytic mixtures containing gold nanorods functionalized with PTEBS (left), PSS (middle) and MUS (right). Experimental conditions: [Au0] = 0.10 mM, [NAD^+] = 1 mM, TEAOH = 0.75 M, temperature = 35 °C, spectral range of incident light = 450-1200 nm, power density 120 mW/cm^2.

4. *The authors should be lauded for discussing possible photothermal heating that may lead to non-Arrhenius kinetics and provide a possible alternative explanation for the observed catalytic effect. The discussion on page 11ff is detailed but loses, in my opinion, the connection to the original question. How likely is it that thermal effects (rather than plasmonic catalysis) are dominating the catalysis? A pointed conclusion would make the manuscript more readable and possible more convincing. If the authors do not want to take a clear stance, they should simply write this (they already do point out possibly experimental routes to a more detailed answer.)*

We do agree with the reviewer. The paragraph on page 11 does not support the central message of the work. But we would like to stress that providing a short discussion on an alternative mechanism (thermal effect) is necessary since we do not have experimental evidence on the change of the local temperature under light irradiation.

Marek Grzelczak
CSIC Research Scientist
Center for Materials Physics CSIC-UPV/EHU
Manuel Lardizabal Ibilbidea 5,
20018 Donostia - San Sebastian
Phone: +34 943 018971
Email: marek.g@csic.es

To answer the reviewer's question, we have rewritten the paragraph in which we made the reader aware of possible collective heating. We have also made a brief projection for follow-up studies by pointing out that in our hybrid system, the conjugated polymer emits detectable fluorescent decay light (Figure 2d). Thus, by taking advantage of the temperature-dependent lifetime of the excited states, the system can be used to measure the in-situ temperature change during the photocatalytic process.

The new text reads as follows (Page 13):

> "Thus, since ζm << 1, the collective heating under steady-state radiation can accelerate the oxidation of TEAOH to glycolaldehyde close to the particles surface followed by NADH regeneration at larger distances. However, to monitor the local temperature change, it is necessary to use sophisticated methods such as, for instance, fluorescent thermometry combined with DNA technology.[38] Note that in our hybrid system, emission from the polymer shell makes possible measurements of the lifetime of excited states (Figure 2d), which in principle is sensitive to the local temperature. Therefore, we postulate that the present system makes it possible to monitor the change of temperature in-situ during a photocatalytic process."

5. *Typo: "nm" is missing after 340 on page 10.*

We apologies for this mistake. The text has been corrected accordingly.

REVIEWER # 2:

In this work, Rogolino et al report a water-soluble metal-polymer heterojunction (Au@PTEBS) in colloidal-phase, which is used in plasmonic catalysis for NADH regeneration. The in-situ spectroscopy, ex-situ advanced electron microscopy, molecular dynamics, and DFT calculations, et al., have been used to reveal the relationship between the polymer molecules and the metal surface. Furthermore, the photocatalytic activity of Au@PTEBS was tested in the light-assisted photoreduction of NAD^+ to NADH using triethanolamine (TEAOH) as an electron donor. The work is solid and meaningful and therefore it can be considered for publication in prestigious The Journal of Physical Chemistry Letters after addressing the next remarks:

1. *There are a lot of contents discussed about the material of the metal-polymer heterojunction in the "Abstract and Conclusion" section, but which is seldom focus on the NADH regeneration performance study. Please added the relative discussion.*

We thank the reviewer for this comment. To strike a balance between material characterization and photocatalytic process in the text, we have modified the abstract and summary section.

Abstract (Page 2):

Marek Grzelczak
CSIC Research Scientist
Center for Materials Physics CSIC-UPV/EHU
Manuel Lardizabal Ibilbidea 5,
20018 Donostia - San Sebastian
Phone: +34 943 018971
Email: marek.g@csic.es

> “Plasmonic catalysis in the colloidal phase requires robust surface ligands that prevent particles from aggregation in adverse chemical environments and allow carrier flow from reagents to nanoparticles. This work describes the use of a water-soluble conjugated polymer comprising thiophene moiety as a surface ligand for gold nanoparticles to create a hybrid system that, under the action of visible light, drives the conversion of the bio-relevant NAD^+ to its high energetic reduced form NADH. A combination of advanced microscopy technique and numerical simulations revealed that the robust metal-polymer heterojunction, rich in sulfonate functional groups, directs the interaction of electron donor molecules with plasmonic photocatalyst. The tight binding of polymer to the gold surface precludes the need for conventional transition metal surface co-catalysts, which were previously shown essential for photocatalytic NAD^+ reduction but are known to hinder the optical properties of plasmonic nanocrystals. Moreover, computational studies indicated that the coating polymer fosters a closer interaction between the sacrificial electron donor triethanolamine and the nanoparticles, thus enhancing the reactivity.”

Conclusions section (Page 15):

> “In summary, the rational combination of polymer and plasmonic particles leads to a hybrid structure in which both components are mutually affected: gold nanocrystals experience strong electron doping, while the polymer particles exhibit faster radiative recombination. For the sake of generality, we showed that polymer can stabilise gold nanoparticles of different shapes that have a cationic surfactant as the native ligand. Detailed structural analysis revealed that the polymer molecules form a homogeneous shell on the surface of gold nanorods and the structuring of the polymer on the nanoparticles surface is an important ingredient in the design of photocatalytic systems, favouring efficient oxidation of sacrificial molecules. As a proof of concept, we showed that conjugated polymers could successfully replace noble metal co-catalysts in the regeneration of the ubiquitous cofactor NADH. In particular, hydrophilic moieties of polythiophene favour the interaction between the plasmonic nanoparticles and the widely used electron donor triethanolamine. The observed enhanced electron transfer in metal-polymer heterojunction opens new possibilities in plasmon-assisted reductive catalysis.”

2. *Figures in the manuscript should be appeared in the normal order. For example, Figure 3d should be discussed after Figure 3a. Please adjust the sequence of the Figure or the modify the relative contents.*

We thank reviewer for this comment. On page 6, we have modified the text referencing the results on Figure 3 and changed the labels order in Figure 4. We are convinced that in the present stage the readability of the text is improved.

3. *Please discussed the stability of the metal-polymer heterojunction in the manuscript. Such as the NADH regeneration experiments on cyclic stability tests should be added.*

Marek Grzelczak
CSIC Research Scientist
Center for Materials Physics CSIC-UPV/EHU
Manuel Lardizabal Ibilbidea 5,
20018 Donostia - San Sebastian
Phone: +34 943 018971
Email: marek.g@csic.es

We thank the reviewer for this comment. We have performed additional experiments for cyclic regeneration of cofactor and we have included the data in Figure 4g (see the figure above in the answer to question #3 of the reviewer #1).

A corresponding discussion has been added into the manuscript on page (Page 12):

> "Also, a large number of anchoring points in PTEBS polymer ensures the structural integrity of the hybrid system, as confirmed by NADH photoregeneration in five consecutive cycles (Figure 4g and Figure S7). A drop in performance with each cycle was due to the losses of nanoparticles in each centrifugation step."

We have also added a new Figure into the supporting information (page 15):

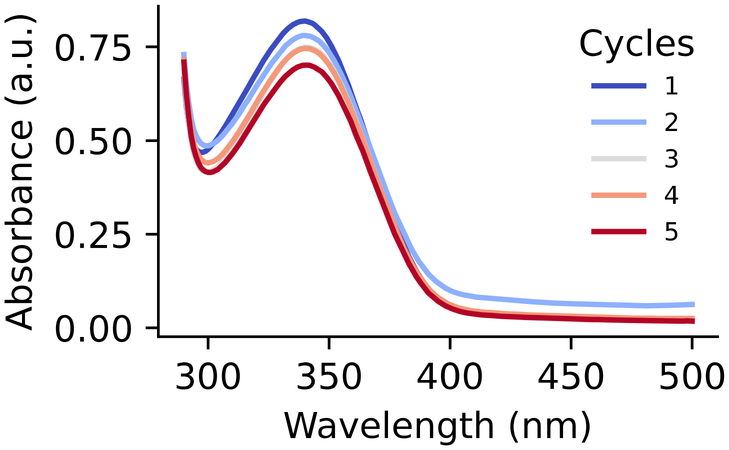

Figure S7: UV-vis spectra of centrifuged mixtures after each cycle of photocatalytic run. Experimental conditions: [Au^0] = 0.10 mM, [NAD^+] = 1 mM, TEAOH = 0.75 M, temperature = 35 °C, spectral range of incident light = 450-1200 nm, power density 120 mW/cm^2.

4. *All Journal names should be abbreviated and unified in the References part. Some Journal names have been wrong, please corrected them.*

We have edited reference formatting. The journals acronyms and format of the tiles have been corrected.

5. *Please supply the full English name of PEDOT in the manuscript.*

We have added the full name of PEDOT (Page 15):

> "For example, the combination of water-soluble poly(3,4-ethylenedioxythiophene) (PEDOT) [46] with plasmonic nanoparticles isan attractive strategy for constructing p-n junction architectures down to the level of a few nanoparticles."

Marek Grzelczak
CSIC Research Scientist
Center for Materials Physics CSIC-UPV/EHU
Manuel Lardizabal Ibilbidea 5,
20018 Donostia - San Sebastian
Phone: +34 943 018971
Email: marek.g@csic.es

6. *Please modify the graphical TOC and supply a beautiful picture.*

We have modified TOC images accordingly.

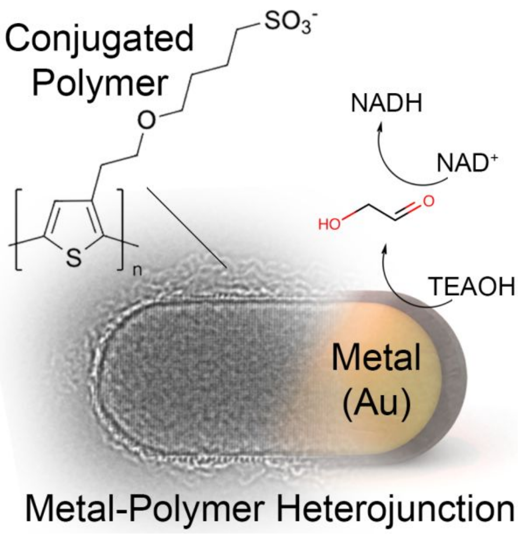

Figure. TOC image

7. *Hydrophilic and hydrophobic of the polymer plays an important role in the metal surface as well as the catalysis, please discussed it in detail in the manuscript.*

We thank the reviewer for this comment. Indeed, the effect of hydrophobicity in the water-soluble conjugated polymer can have an important impact on the ligand exchange process and also on the catalytic performance. We have introduced the following discussion at the end of the last paragraph (Page 15):

> "Therefore, the amphiphilic nature of PTEBS has a beneficial role in terms of colloidal stabilization and catalysis: i) hydrophobic thiophene moieties strongly binds to the surface of nanoparticles replacing CTAB and preventing aggregation, ii) hydrophilic sulfonate groups bear negative charges to the surface, as indicated by the reversed value of zeta-potential after ligand exchange, which is likely to improve the docking of triethanolamine on Au and subsequent oxidation to glycolaldehyde."
